# Activation of ACLY by SEC63 deploys metabolic reprogramming to facilitate hepatocellular carcinoma metastasis upon endoplasmic reticulum stress

**DOI:** 10.1186/s13046-023-02656-7

**Published:** 2023-05-01

**Authors:** Chenyu Hu, Zechang Xin, Xiaoyan Sun, Yang Hu, Chunfeng Zhang, Rui Yan, Yuying Wang, Min Lu, Jing Huang, Xiaojuan Du, Baocai Xing, Xiaofeng Liu

**Affiliations:** 1 Hepatopancreatobiliary Surgery Department I, Key Laboratory of Carcinogenesis and Translational Research (Ministry of Education/Beijing), Peking University Cancer Hospital & Institute, Beijing, 100142 People’s Republic of China; 2 Department of Medical Genetics, School of Basic Medical Sciences, Peking University Health Science Center, Beijing, 100191 People’s Republic of China; 3 Department of Genetics, Harvard Medical School, Boston, 02115 USA; 4 Department of Cell Biology, School of Basic Medical Sciences, Peking University Health Science Center, Beijing, 100191 People’s Republic of China; 5 Department of Pathology, School of Basic Medical Sciences, Peking University Health Science Center, Beijing, 100191 People’s Republic of China; 6 Department of Immunology, School of Basic Medical Sciences, Peking University, and NHC Key Laboratory of Medical Immunology (Peking University), Beijing, 100191 People’s Republic of China

**Keywords:** ER stress, Metabolic reprogramming, Hepatocellular carcinoma, Metastasis, ACLY, SEC63

## Abstract

**Background:**

Tumor cells display augmented capability to maintain endoplasmic reticulum (ER) homeostasis and hijack ER stress pathway for malignant phenotypes under microenvironmental stimuli. Metabolic reprogramming is a well-known hallmark for tumor cells to provide specific adaptive traits to the microenvironmental alterations. However, it’s unknown how tumor cells orchestrate metabolic reprogramming and tumor progression in response to ER stress. Herein, we aimed to explore the pivotal roles of SEC63-mediated metabolic remodeling in hepatocellular carcinoma (HCC) cell metastasis after ER stress.

**Methods:**

The expression levels of SEC63 in HCC tissues and adjacent non-cancerous tissues were determined by immunohistochemistry and western blot. The regulatory roles of SEC63 in HCC metastasis were investigated both in vitro and in vivo by RNA-sequencing, metabolites detection, immunofluorescence, and transwell migration/invasion analyses. GST pull-down, immunoprecipitation/mass spectrometry and in vivo ubiquitination/phosphorylation assay were conducted to elucidate the underlying molecular mechanisms.

**Results:**

We identified SEC63 as a new regulator of HCC cell metabolism. Upon ER stress, the phosphorylation of SEC63 at T537 by IRE1α pathway contributed to SEC63 activation. Then, the stability of ACLY was upregulated by SEC63 to increase the supply of acetyl-CoA and lipid biosynthesis, which are beneficial for improving ER capacity. Meanwhile, SEC63 also entered into nucleus for increasing nuclear acetyl-CoA production to upregulate unfolded protein response targets to improve ER homeostasis. Importantly, SEC63 coordinated with ACLY to epigenetically modulate expression of Snail1 in the nucleus. Consequently, SEC63 promoted HCC cell metastasis and these effects were reversed by ACLY inhibition. Clinically, SEC63 expression was significantly upregulated in HCC tissue specimens and was positively correlated with ACLY expression. Importantly, high expression of SEC63 predicted unfavorable prognosis of HCC patients.

**Conclusions:**

Our findings revealed that SEC63-mediated metabolic reprogramming plays important roles in keeping ER homeostasis upon stimuli in HCC cells. Meanwhile, SEC63 coordinates with ACLY to upregulate the expression of Snail1, which further promotes HCC metastasis. Metastasis is crucial for helping cancer cells seek new settlements upon microenvironmental stimuli. Taken together, our findings highlight a cancer selective adaption to ER stress as well as reveal the potential roles of the IRE1α-SEC63-ACLY axis in HCC treatment.

**Supplementary Information:**

The online version contains supplementary material available at 10.1186/s13046-023-02656-7.

## Background

Endoplasmic reticulum (ER) plays pivotal roles in protein folding, modifications and trafficking. The ER homeostasis is highly sensitive to environmental stimuli such as hypoxia, denutrition and oncogenic activation. These stimuli decrease the efficacy of ER, leading to an accumulation of misfolded or unfolded proteins in the ER lumen, an event called ER stress [1]. Usually, the misfolded proteins are unable to leave the ER and may eventually actuate a signaling cascade defined as the unfolded protein response (UPR) through three branches including protein kinase RNA-like ER kinase (PERK), inositol-requiring enzyme 1 (IRE1α), and activating transcription factor 6 (ATF6) pathways [2]. The initiation of UPR will try to lighten the burden of ER by decelerating protein translation or by increasing the ER’s folding capacity to support cell survival. However, continuous or severe ER stress will cause the activation of pro-apoptotic pathway.

Rapid proliferation of tumor cells requires an acute increase of protein synthesis, thereby inevitably causing the UPR [3]. Moreover, solid tumor cells often suffer from diverse microenvironmental perturbations. Therefore, the UPR are frequently induced in nearly all cancer types and ER stress pathways seem to affect every hallmark of cancer [4, 5]. Cancer cells can hijack ER stress pathways for supporting the malignant activities. Dysregulated UPR signaling is often accompanied by upregulated tumor cell growth, invasion capacity and drug-resistance [3, 6]. However, the underlying mechanisms by which tumor cells hijack ER stress have not been fully elucidated.

Metabolic remodeling is a well-known hallmark for tumor cells to accommodate the biosynthetic pathways as well as provide specific adaptive traits to the microenvironmental stimuli [7–9]. Acetyl-CoA is one of the central metabolic intermediates. Derived from multiple pathways of catabolism, Acetyl-CoA is not only the raw material for lipid biosynthesis to uphold the cellular membrane system but also the substrate for protein acetylation reactions to epigenetically modulate gene expression [10, 11]. Therefore, the cellular acetyl-CoA pool is critical for a variety of biological activities such as cell growth, mitosis, or autophagy [12]. In mammals, acetyl-CoA is mainly produced from glycolysis, β-oxidation of fatty acids, and glutamine decomposition [13]. Especially, tumor cells utilize acetate as a substitutable carbon source for generating acetyl-CoA [14]. The critical enzymes for synthesizing acetyl-CoA, including adenosine triphosphate (ATP)-citrate lyase (ACLY), acetyl-CoA synthetase 1 (ACSS1), and acetyl-CoA synthetase 2 (ACSS2), are observed to have important effects in promoting tumor progression [14–16]. Nevertheless, it’s still undetermined whether cancer cells orchestrate acetyl-CoA metabolic reprogramming to maintain malignant state under ER stress.

Hepatocellular carcinoma (HCC), the most frequent primary liver cancer, is the second leading cause of cancer-related death in male worldwide [17]. Despite various management regimens that have been applied in HCC treatment, the prognosis of HCC is very dismal owing to the high probability of metastasis [18, 19]. Tumor cell metabolic reprogramming is tightly associated with HCC progression [20]. Thus, the deep understandings of the mechanisms underlying HCC cell metabolic reprogramming contribute to uncover effective approaches for HCC treatment.

SEC63, as a component of active post-translational Sec translocon, mainly locates at ER and participates in protein trafficking [21]. The germline mutations of *SEC63* gene are associated with polycystic liver disease [22]. However, the role of SEC63 in HCC progression has not been elucidated. Here, we identified a new critical pathway by which HCC cells hijack ER stress to enhance metastasis. Mechanistically, phosphorylation of SEC63 at T537 improves ACLY stability to orchestrate metabolic and epigenetic reprogramming for metastasis after ER stress. Our findings indicate the key roles of SEC63 in HCC metastasis and imply its potential as a therapeutic target for HCC treatment.

## Methods

### Patients and specimens

We analyzed 139 patients who underwent curative hepatectomy as initial treatment for HCC at Peking University Cancer Hospital (PUCH) between 2009 and 2011. Patients with other malignancies, absence of paraffin-embedded clinical tissue specimens and with incomplete clinicopathological information were excluded. Collection of the HCC tissues and adjacent non-tumor tissues was approved by the Ethics Committee of Peking University Cancer Hospital & Institute. Written informed consent was obtained from the participating patients and the relevant clinical and histopathological data provided to the researchers were anonymized. Tumor staging was performed using the Barcelona Clinical Liver Cancer (BCLC) staging system. Clinical information regarding the samples is presented in Supplementary Table S1.

### Cell culture and reagents

HCC cell lines were maintained in DMEM medium or RPMI 1640 medium. All medium was supplemented with 10% fetal bovine serum and 1% antibiotic solution. All cell lines were cultured in 5% CO_2_ at 37 °C in incubators with 100% humidity. The antibodies and reagents for this study are listed in Supplementary Table S2.

### Cell transfection or infection

Cells were transfected with plasmid DNA or siRNA RNA duplexes by Lipofectamine 2000 (Invitrogen) according to the manufacturer’s protocol. In transient transfection experiments, plasmid DNA was kept constant with empty vector. To construct stable SEC63-overexpressed cell lines, pLV-EGFP-SEC63 or pLV-EGFP empty vector were transfected into HEK293T cells with the packaging vectors pH1 and pH2 to produce lentiviral particles. To construct stable SEC63 knockdown cell lines, pLKO.1-SEC63 shRNA or pLKO.1-Ctrl shRNA were transfected into HEK293T cells with the packaging vectors pMD2.G and psPAX2 to produce lentiviral particles. HCC cells were infected with lentiviruses delivering pLV-EGFP-SEC63 or SEC63 shRNA to obtain stable SEC63 overexpression and knockdown cell lines. Short interfering RNA (siRNA) sequences were directly synthesized (GenePharma, Shanghai, China). The sequences of shRNAs and siRNAs are listed in the Supplementary Table S3.

### Plasmids

Flag-tagged SEC63, SMAD3 and SEC63 mutants (S1, S2, S3, delN, T537A, T537E) were cloned into pCI-neo or pCMV vector. Similarly, Flag-tagged ACLY or its mutants (A1, A2, A3, A4) were cloned into pCI-neo vector. Flag-tagged Snail1was cloned into pCI-neo vector. GFP-tagged SEC63 was cloned into pLV-EGFP vector. GFP-tagged SEC63 delN was cloned into pEGFP-C2 vector. For in vitro glutathione S-transferase (GST) pull-down assay, ACLY mutants (A3, A4) were cloned into pGEX-4 T-1 vector. His-tagged SEC63 S3 was cloned into pET28b. All PCR products were confirmed by DNA sequencing. Point mutant plasmids were constructed by mutagenesis using the Fast Site-Directed Mutagenesis Kit (TIANGEN) according to the manufacturer’s protocol. The presence of the mutations in plasmids was confirmed by DNA sequencing.

### Western blot analysis

Total proteins were extracted from tissue samples or HCC cell lines and were separated by electrophoresis. Then, the proteins were transferred onto polyvinylidene fluoride membranes (Amersham Biosciences). Blots were hybridized with the indicated antibodies after being blocked with 5% milk. After washing with PBS/T, blots were incubated with IRDye® 680RD Goat anti-Rabbit/Mouse IgG (H + L) secondary antibodies. Immunocomplexes were detected using the LiCor Odyssey CLx imager.

### Coimmunoprecipitation (Co-IP) assay and mass spectrometry

Cells were harvested and cell lysates were prepared in Buffer A (25 mM Tris–Cl pH 7.5, 150 mM KCl, 1 mM DTT, 2 mM EDTA, 0.5 mM PMSF, and 0.2% Nonidet P-40) and used for immunoprecipitation. The indicated antibodies were coupled with 50% suspension of protein A-Sepharose beads (GE Healthcare) in Buffer IPP500 (500 mM NaCl, 10 mM Tris–Cl pH 8.0, and 0.2% Nonidet P-40). Coupled beads were incubated with cell lysates for 2 h at 4 °C. After washing, the precipitates were examined by western blot using the indicated antibodies. Mass spectrometry was performed at the Institute of Biotechnology of Peking University Health Science Center (Beijing, China).

### Transwell migration and invasion assay

Cell migration and invasion assays were carried out in 24-well transwell chambers with 8-μm-pore polycarbonate filter inserts (Corning Inc., New York, USA). Briefly, HepG2, Huh7 or PRF/PLC/5 cells were seeded in uncoated or matrigel-coated (BD Biosciences) inserts. The lower chambers were filled with 10% FBS-supplemented DMEM as chemoattractant. Then, cells on the upper side of the filter were removed and those on the lower surface of the insert were fixed in 4% paraformaldehyde and stained with crystal violet (Beyotime Biotechnology, Shanghai, China). The migrated or invaded cells were quantified by ImageJ software.

### Wound healing assay

Cells were transfected as indicated and seeded into 6-well plates. When reaching 90% cell confluence, a straight line was drawn on the cell monolayer. After washing the cell debris with PBS, cells were incubated with serum-free DMEM at 37 °C. Images of migratory cells were captured at indicated time points under a light microscope. The relative cell migration rate was analyzed using ImageJ software.

### Metabolite measurements

Cells seeded in 10 cm dishes were grew to 80% of confluence and used for the measurements of various metabolites. For acetyl-coA measurement, cells were washed with cold PBS and harvested in Acetyl-CoA assay buffer provided by Acetyl-CoA assay kit, and then the acetyl-CoA concentration was determined using Acetyl-CoA Assay Kit (AK340, Bioss, Beijing, China) according to the manufacturer’s instructions. For cholesterol measurement, cells were harvested using isopropanol and then sonicated. After centrifugation, the supernatant was used to determine total cholesterol levels using a Total Cholesterol Levels Assay Kit (BC1985, Beijing Solarbio Science & Technology Co. LTD, China) according to manufacturer’s instructions. For triglyceride measurement**,** cells were treated with n-Heptane: isopropanol (1:1), and then sonicated. After centrifugation, the supernatant was used to determine triglyceride levels using a Triglyceride Levels Assay Kit (BC0620, Beijing Solarbio Science & Technology Co. LTD, China) according to manufacturer’s instructions. For free fatty acid measurement, cells were treated with n-Heptane: absolute methanol: chloroform (24:1:25) and centrifugated, the supernatant was used to determine free fatty acid levels using a Free Fatty Acid Levels Assay Kit (BC0590, Beijing Solarbio Science & Technology Co. LTD, China) according to manufacturer’s instructions.

### Immunohistochemistry (IHC) assay

Tissue sections were de-paraffinized in xylene and rehydrated in a graded ethanol series. To inactivate endogenous peroxidase activity, the sections were treated using a 0.3% peroxidase solution for 30 min. The sections were boiled with citrate buffer for 15 min at 100 °C for antigen retrieval. After treatment with blocking buffer, the sections were incubated with a primary antibody at 4 °C overnight. Next, the sections were visualized using an UltraVision Quanto Detection System HRP DAB Kit (ZSGB-Bio, China) according to the manufacturer’s protocols. The sections were counterstained with hematoxylin and were evaluated by pathologists blinded to the clinical outcome. The percentages of positive tumor cells were scored as follows: 1%, 0 points; 1–25%, 1 point; 26–50%, 2 points; 51–75%, 3 points; and 75%, 4 points. The staining intensity was scored as follows: no staining, 0 points; weak staining, 1 point; moderate staining, 2 points; and strong staining, 3 points. Then, the two scores were multiplied to acquire a combined score ranging from 0 to 12.

### Immunofluorescence (IF) staining

Cells were fixed with 4% paraformaldehyde for 15 min and permeabilized using 0.2% Triton X-100 for 10 min at room temperature. After blocking with 10% goat serum, the cells were incubated with primary antibodies overnight at 4 °C. After washing with PBS, an FITC-conjugated anti-rabbit antibody and a TRITC-conjugated anti-mouse antibody were added, and the samples were incubated for 1 h at room temperature. Finally, the cells were stained with DAPI to visualize the nuclei.

### Quantitative real-time PCR (RT-qPCR) assay

Total RNAs were extracted from cells using TRIzol reagent (Invitrogen, USA), purified using the RNeasy Mini Kit (Qiagen, Germany) according to the manufacturer’s instructions, and electrophoresed on a denaturing agarose gel to examine the RNA integrity. Then, cDNA was synthesized from 2 μg of total RNA using the Superscript First-Strand Synthesis System (Invitrogen), and qPCR was performed according to Minimal Information for Publication of Quantitative Real-Time PCR Experiments (MIQE) guidelines. The sequences of the qPCR primers are shown in Supplementary Table S4.

### Chromatin immunoprecipitation (ChIP) assay

ChIP assay was performed using a Pierce Magnetic ChIP Kit (Cell Signaling Technology). Briefly, formaldehyde was added to the cells to cross-link nuclear proteins with genomic DNA, and glycine (0.125 M) was added to stop the cross-linking. The cells were collected, centrifugated and resuspended in FA lysis buffer (1% SDS, 10 mM EDTA, protease inhibitors and 50 mM Tris–HCl pH8.0) and genomic DNA was sonicated to get a length of approximately 200 to 1000 bp. Cell lysates were clarified by centrifugation, diluted by 1:10 in ChIP dilution buffer (0.01% SDS, 1.0% Triton X-100, 1.2 mM EDTA, 16.7 mM NaCl, protease inhibitors and 16.7 mM Tris–HCl pH 8.0) and incubated with anti-acetyl-H3 or control IgG overnight at 4 °C with rotation. ChIP-Grade Protein A/G Magnetic Beads (Cell Signaling Technology) were added to each reaction and incubated for two hours. Protein A/G magnetic beads were pelleted by centrifugation and washed with the following buffers: low salt wash buffer (0.1% SDS, 1% Triton X-100, 2 mM EDTA, 150 mM NaCl and 20 mM Tris–HCl pH 8.0), high salt wash buffer (0.1% SDS, 1% Triton X-100, 2 mM EDTA, 500 mM NaCl and 20 mM Tris–HCl pH 8.0) and LiCl wash buffer (0.25 mM LiCl, 1% NP-40, 1% sodium deoxycholate, 1 mM EDTA and 10 mM Tris–HCl pH 8.0). Finally, beads were washed twice with 1 ml of TE buffer (1 mM EDTA and 10 mM Tris–HCl pH 8.0). The protein-DNA cross-linking was reversed by incubation with 200 mM NaCl and proteinase K for 2 h at 65 °C. DNA was extracted and subjected to real-time qPCR reaction. The input DNA and IgG were used for normalization and the primers are listed in Supplementary Table S4.

### GST pull-down assay

GST fusion proteins were obtained according to the standard protocol. For in vitro binding assays, GST fusion proteins bound to the Glutathione Sepharose 4B were incubated with purified proteins. Then, the bound proteins were separated by electrophoresis and immunoblotted with indicated antibodies.

### RNA sequencing and bioinformatic analysis

Total RNA from control or SEC63-depleted Huh7 cells was isolated to perform RNA sequencing (RNAseq), which was carried out by ANNOROAD (Beijing, China). The differentially expressed genes were subjected to protein–protein interaction (PPI) analysis using the STRING (search tool for recurring instances of neighbouring genes). The hub genes were selected by Cytohubba plug-in using Cytoscape software.

### Animal study

Female BALB/c nude mice at 6 weeks of age were purchased from Beijing Vital River Laboratory Animal Technology Co., Ltd. (Beijing, China). For the experimental mouse lung metastasis model, 1 × 10^6^ Huh7 cells were injected into nude mice through the tail vein (*n* = 6, per group). Drug treatment protocols included intraperitoneal injection with physiological saline (control group), and ETC1002 (30 mg/kg per 2 days for 8 weeks). Mice were sacrificed after 8 weeks and lungs were removed, fixed in paraformaldehyde (4%), and embedded in paraffin. Consecutive sections were made for every lung tissue block and stained with hematoxylin and eosin. The number of lung metastases was counted and evaluated independently by two pathologists. All animal experiments were reviewed and approved by the Ethics Review Committee of Peking University Health Science Center.

### Database

The RNA sequencing data and the related clinical data of HCC were obtained from TCGA website (https://portal.gdc.cancer.gov/) (TCGA-LIHC dataset) or were collected from the ICGC database (https://dcc.icgc.org/) (ICGC-LIRI-JP dataset). GSE101728 or GSE124535 datasets were download from the GEO website (https://www.ncbi.nlm.nih.gov/geo/).

### Statistical analyses

The significance of the differences was analyzed via one-way ANOVA or two-tailed student’s t-test. Spearman’s correlation coefficient was employed to calculate the correlations between the two groups. Kaplan–Meier analysis was used for survival analysis and the differences in the survival probabilities were calculated using the log-rank test. The univariate and multivariate analysis of prognostic factors were conducted using the Cox regression model. The statistical analyses were performed using GraphPad Prism or SPSS version 17.0 (SPSS, Inc.). *P* < 0.05 was considered to indicate statistical significance. *P < 0.05, **P < 0.01, ***P < 0.001, and ****P < 0.0001 for all the analyses.

## Results

### Overexpression of ACLY is correlated with poor outcomes of HCC patients

**Fig. 1:**
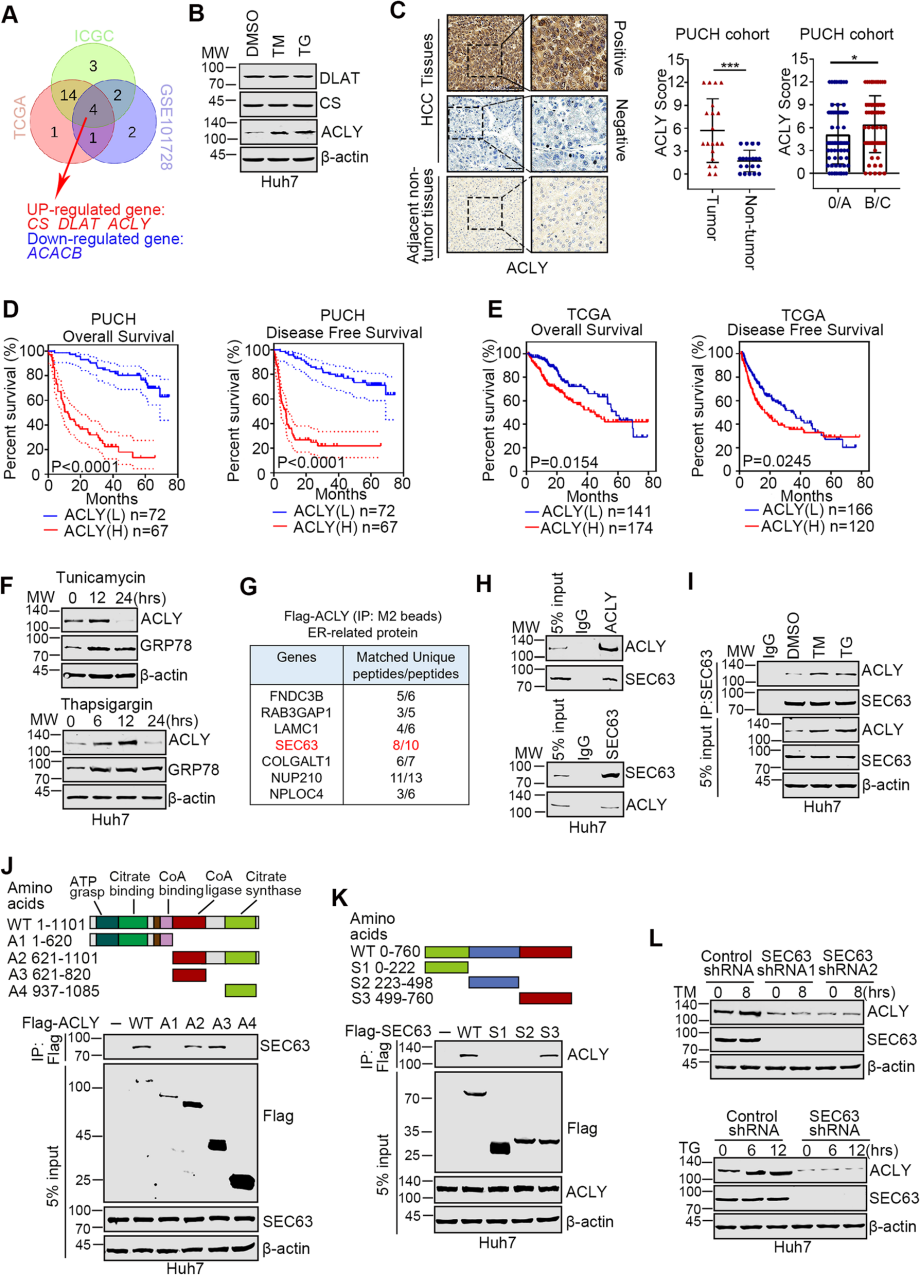
ACLY is upregulated by SEC63 in response to ER stress in HCC cells. **A** Venn diagrams of overlapped differentially expressed Acetyl-CoA-Related Genes (ARGs) among TCGA-LIHC, ICGC-LIRI-JP and GSE101728 cohorts. **B** Huh7 cells were treated with tunicamycin (TM, 5 μg/mL), or thapsigarin (TG, 1 μM) for 4 h. Then, the indicated proteins were detected by western blot. **C** Representative IHC staining of ACLY in HCC and adjacent non-tumorous tissues. Summary of ACLY expression in 139 cases of HCC and 20 cases of non-tumorous counterparts. Scale bars, 50 μm. **D** The overall survival (OS) or disease-free survival (DFS) of patients with HCC exhibiting different levels of ACLY staining was determined by Kaplan–Meier analysis. **E** The survival curves of the HCC patients with different ACLY mRNA levels from TCGA-LIHC cohort were calculated. **F** Huh7 cells were treated with TM (5 μg/mL) or TG (1 μM) for the indicated time points. Then, the expression of ACLY was evaluated by western blot. **G** The ER-related proteins among ACLY-interacting partners were identified by Co-IP-mass spectrometry. **H** Huh7 cell lysates were immunoprecipitated with anti-SEC63 or anti-ACLY antibody. Immuno-precipitates were immunoblotted as indicated. **I** Huh7 cells were treated with TM (5 μg/mL) or TG (1 μM) for 8 h and immunoprecipitation was performed with anti-SEC63 antibody. **J, K** Huh7 cells were transfected with ACLY or SEC63 deletion mutants and treated with MG132. Co-IP was performed using the anti-Flag antibodies. **L** The control and SEC63-depleted cells were treated with TM (5 μg/mL) or TG (1 μM) for the indicated time points, and the expression of ACLY and SEC63 was detected by immunoblotting

To explore Acetyl-CoA metabolic reprogramming upon ER stress in HCC cells, we analyzed the expression patterns of acetyl-CoA metabolism-related genes (ARGs) in HCC tissues and adjacent non-cancerous tissues from three HCC cohorts, including TCGA-LIHC, ICGC-LIRI-JP and GSE101728 (Supplementary Fig. 1A). The differentially expressed ARGs from each dataset were combined in a Venn diagram, and the overlapping genes were identified (Fig. 1A). Among the overlapping genes, *DLAT*, *CS* and *ACLY* were upregulated in cancerous tissues while *ACACB* was downregulated in tumorous tissues compared to adjacent non-tumorous tissues (Supplementary Fig. 1B). Next, we used Thapsigargin (TG) and Tunicamycin (TM) as the inducer of ER stress to mimic adverse microenvironmental stimuli in tumor cells [6] and evaluated the expression level of the upregulated genes. As shown in Fig. 1B, only ACLY was upregulated after ER stress. These data suggest that ACLY might play critical roles in regulating HCC cell homeostasis upon ER stress. To analyze the clinical relevance of ACLY in HCC, we detected ACLY expression in 139 cases of HCC samples and the patients were divided into two groups (ACLY Low or ACLY High expression). ACLY was increased in the tumor tissues compared with non-tumorous tissues and upregulated at stage B/C (Fig. 1C). Survival analysis revealed that ACLY high expression predicted shorter overall survival (OS) and disease-free survival (DFS) time compared to ACLY low expression group (Fig. 1D). Consistently, data analysis from TCGA cohort or ICGC-LIRI-JP cohort also revealed that ACLY overexpression was correlated with poor prognosis of HCC patients (Fig. 1E and Supplementary Fig. S1C).

### ACLY is upregulated by SEC63 in response to ER stress

We further analyzed the expression levels of ACLY in HCC cells treated with thapsigargin or tunicamycin. As shown in Fig. 1F, GRP78, one of ER stress markers [23], was significantly increased, suggesting the successful induction of ER stress. Moreover, ACLY was upregulated after treatment with either TM or TG for a short time, which is similar with GRP78. However, ACLY was reduced when cells were treated for a long period (24 h) with the inducing drugs, suggesting that severe ER stress failed to upregulate ACLY. Since it’s well known that excessive ER stress induced cell death [24], we next determined the underlying mechanism by which ACLY is activated upon primitive ER stress. Co-immunoprecipitation (Co-IP) and mass spectrometry were performed to uncover latent binding proteins of ACLY related to ER stress. Localization analysis revealed that seven proteins were ER-related proteins (Fig. 1G). Among these proteins, SEC63 was of particular interest because of its role in regulating ER stress [25]. Co-IP assays showed that SEC63 indeed interacted with ACLY (Fig. 1H), and the interaction was enhanced by ER stress (Fig. 1I). The immunofluorescent staining showed that ACLY and SEC63 were mainly co-localized in the cytoplasm in HCC cells (Supplementary Fig. S1D). ACLY contains five structural domains: ATP-grasp, Citrate binding, CoA binding, CoA ligase, and Citrate synthase [26]. The SEC63-binding region of ACLY was narrowed down to the CoA-ligase domain by Co-IP experiments (Fig. 1J). Further, the C-terminus of SEC63 was required for the interaction with ACLY (Fig. 1K). Moreover, GST pull-down experiment performed with purified proteins confirmed that the C-terminus of SEC63 specifically bound to GST-CoA ligase domain (Supplementary Fig. S1E). Finally, ER stress failed to induce ACLY in SEC63-depleted cells (Fig. 1L), indicating that SEC63 is required for activated ACLY under ER stress. Together, these results suggest that ACLY is upregulated by SEC63 in response to ER stress.

### SEC63 is phosphorylated at T537 upon ER stress

**Fig. 2:**
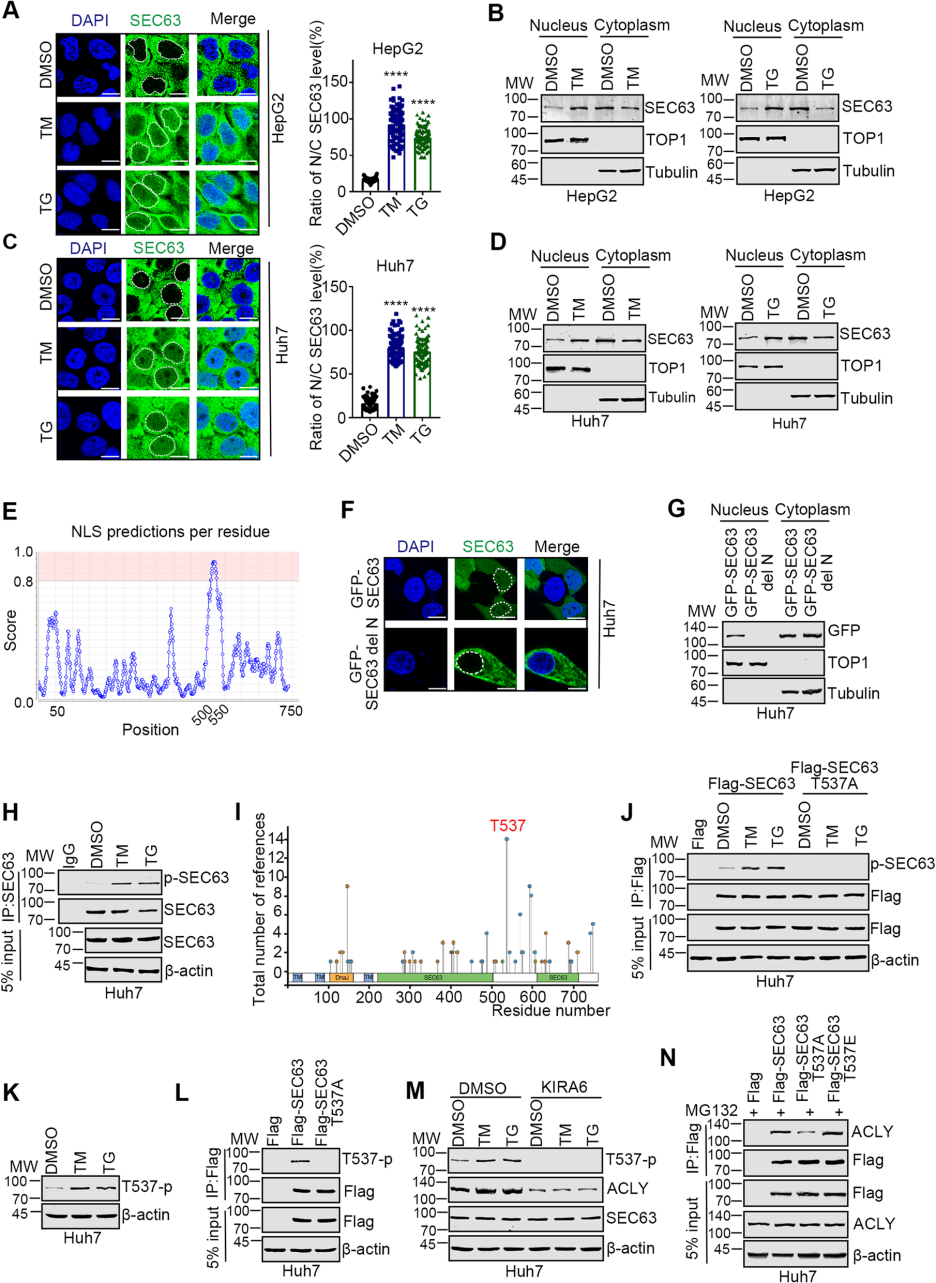
SEC63 is phosphorylated at Thr537 after ER stress. **A** HepG2 cells were treated with TM (5 μg/mL) or TG (1 μM) for 8 h. Immunofluorescence was performed with anti-SEC63 antibody. *n* = 100. **B** HepG2 cells were treated with TM (left) or TG (right). Cell fraction was performed and SEC63 was evaluated by western blot. **C** Huh7 cells were treated with TM (5 μg/mL) or TG (1 μM) for 8 h. Immunofluorescence staining was performed. *n* = 100. **D** Huh7 cells were treated with TM (left) or TG (right). Then, cell fraction was performed. **E** The nuclear localization signal sequence of SEC63 was analyzed by JPred4. **F** Huh7 cells were transfected with GFP-SEC63 or GFP-SEC63 delN. Then, cells were treated with TM and immunofluorescence was performed. Scale bar, 10 μm. **G** Huh7 cells were transfected as indicated and treated with TM. Cell fraction was performed and SEC63 was evaluated by western blot. **H** Huh7 cells were treated with TM (5 μg/mL) or TG (1 μM) for 8 h. Cell lysates were subjected to immunoprecipitation and western blot using pan-phos-Serine/Threonine antibody to detect phosphorylation of SEC63. **I** The phosphorylation sites at SEC63 were predicted by PhosphoSitePlus. **J** Huh7 cells were transfected with Flag-SEC63 or Flag-SEC63 T537A and then treated with TM (5 μg/mL) or TG (1 μM) for 8 h. Immunoprecipitation was performed and followed by western blot to evaluate the level of SEC63 phosphorylation. **K** Huh7 cells were treated with TM or TG. Cell lysates were subjected to western blot using anti-phos-T537 (T537-p) SEC63 antibody. **L** Huh7 cells were transfected with Flag, Flag-SEC63 or Flag-SEC63 T537A. Western blot was performed as indicated. **M** Huh7 cells were pretreated with KIRA6 (10 μM) for 1 h, followed by TM (5 μg/mL) or TG (1 μM) for 8 h. Cell lysates were subjected to western blot. **N** Huh7 cells were transfected as indicated. Then, co-immunoprecipitation was performed using the indicated antibody

It’s unknown how SEC63 is activated upon ER stress. ER stress had little effect on the protein level of SEC63 (Supplementary Fig. S2A-B). Strikingly, we observed the nuclear localization of SEC63 and a portion of SEC63 translocated from the cytoplasm to the nucleus in response to ER stress (Fig. 2A-D). Previous studies reported that SEC63 is an ER-resident protein [27]. To further confirm our results, subcellular localization of SEC63 was predicted by COMPARTMENTS [28]. The prediction suggested that SEC63 also localizes in cytosol and nucleus besides ER (Supplementary Fig. S2C). The nuclear localization signal (NLS) was identified by JPred4 [29], indicating that the region of residues 510–546 may be required for nuclear localization of SEC63 (Fig. 2E). We generated the deletion mutant (delN) of SEC63 which lacks the NLS. Indeed, immunofluorescence and western blot analyses show that delN had no nuclear localization under normal conditions or upon ER stress (Fig. 2F-G and Supplementary Fig. S2D). Together, these results suggest that ER stress induces translocation of SEC63 from cytoplasm to nucleus.

It’s of great interest to explore how SEC63 is activated upon ER stress. Since SEC63 binds to IRE1α [25], we speculated that IRE1α phosphorylates SEC63 with its kinase activity. Serine/threonine phosphorylation of SEC63 was detected in cells treated with TM or TG (Fig. 2H), suggesting that SEC63 is phosphorylated after ER stress. The potential phosphorylation site was predicted at Thr537 by PhosphoSitePlus [30] (Fig. 2I). We then created a SEC63 mutant by replacing Thr537 with alanine to abrogate the phosphorylation and found that phosphorylation was detectable in the immuno-precipitates of wild-type (WT) SEC63, but not of SEC63-T537A (Fig. 2J). To further study the role of Thr537 phosphorylation on SEC63 function, we generated T537-phosphorylation specific antibody (designated as “T537-p”) to recognize the T537-phosphorylation. The specificity of T537-p antibody was confirmed by dot blotting (Supplementary Fig. S2E). Accordingly, using T537-p antibody, we also observed that phosphorylation of SEC63 at T537 was increased upon ER stress (Fig. 2K). Moreover, Flag-SEC63, but not Flag-SEC63 T537A was phosphorylated after ER stress (Fig. 2L). To determine whether SEC63 is phosphorylated by IRE1α pathway, we confirmed the interaction between SEC63 and IRE1α (Supplementary Fig. S2F). Furthermore, inhibition of IRE1α by KIRA6 dramatically decreased the ACLY expression level and the phosphorylation of SEC63 at Thr537 (Fig. 2M). These data suggest that SEC63 is phosphorylated at Thr537 upon ER stress.

Notably, Thr537 localizes in NLS of SEC63. Thus, it’s unknown whether phosphorylation affects the translocation of SEC63 from cytoplasm to nucleus. SEC63 T537A cannot translocate into the nucleus while SEC63 T537E, in which Thr537 was replaced by aspartic acid to mimic phosphorylation status, had an enhanced translocation (Supplementary Fig. S2G). Moreover, T537A mutation decreased the interaction of SEC63 and ACLY while T537E mutation increased the interaction (Fig. 2N). Therefore, these results indicate that phosphorylation of SEC63 at Thr537 promotes nuclear location of SEC63 and enhances the interaction between SEC63 and ACLY after ER stress.

### SEC63 improves the stability of ACLY by counteracting KLHL25

**Fig. 3:**
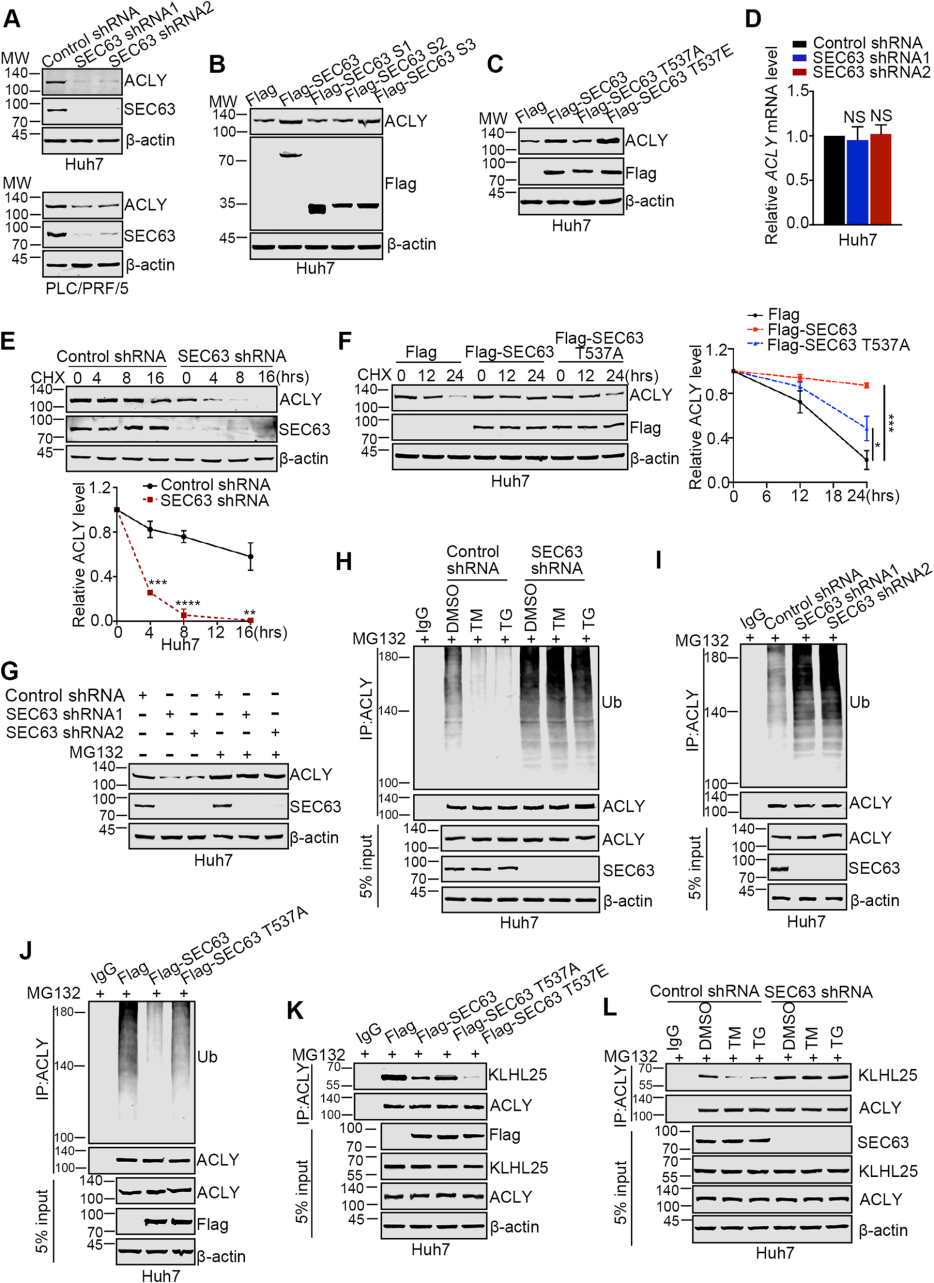
SEC63 increases the stability of ACLY. **A** Whole cell lysates extracted from the Huh7 or PLC/PRF/5 cells were subjected to western blot using anti-SEC63 or anti-ACLY antibodies. **B-C** Huh7 cells were transfected as indicated. Then, western blot was performed. **D** Total RNA extracted from the SEC63-depleted cells or control cells were subjected to RT-qPCR using the ACLY primers. **E** The ctrl cells or SEC63-depleted cells were treated with CHX (50 μg/mL) as indicated time points. Western blot was performed for evaluating ACLY and SEC63 level. ACLY expression level was summarized from three independent experiments (lower panel). **F** Huh7 cells were transfected as indicated and treated with CHX (50 μg/mL) as indicated time points. Western blot was performed for evaluating ACLY and SEC63 level. ACLY expression level was summarized (right panel). **G** The ctrl cells or SEC63-depleted cells were treated with MG132 (10 μM) for 4 h. Western blot was performed as indicated. **H** The ctrl cells or SEC63-depleted cells were treated as indicated. The ubiquitination level of ACLY was analyzed by immunoprecipitation. **I** Huh7 cells were treated with MG132 (10 μM) for 4 h. The ubiquitination level of ACLY was analyzed by immunoprecipitation. **J** After MG132 treatment, the ubiquitination level of ACLY was analyzed by immunoprecipitation in Huh7 cells. **K** Huh7 cells were transfected with indicated plasmids and cell lysates were subjected to Co-IP using anti-ACLY antibody. **L** Ctrl cells or SEC63-depleted cells were treated with the indicated drugs. The cell lysates were subjected to Co-IP using anti-ACLY antibody

Since SEC63 is required for activated ACLY under ER stress, we investigated underlying mechanism regarding to the regulation of ACLY by SEC63. As shown in Fig. 3A, depletion of SEC63 decreased the protein level of ACLY in HCC cells. Similarly, overexpression of SEC63 and SEC63 S3 mutant upregulated ACLY expression level, but SEC63 S1 and S2 mutant failed to do so (Fig. 3B). SEC63 T537A mutant displayed much weaker effect on ACLY expression level while SEC63 T537E mutant showed stronger effect compared with wild-type SEC63 (Fig. 3C), indicating that phosphorylation of SEC63 enhances its function towards ACLY. Moreover, downregulation of SEC63 had no effect on ACLY mRNA level and the mRNA level of ACLY was not changed in response to ER stress (Fig. 3D and Supplementary Fig. S3A), suggesting that SEC63 may modulate ACLY stability per se. To corroborate this hypothesis, we employed cycloheximide (CHX, a ribosome-stalling drug [31, 32]) to block protein translation and analyzed the stability of ACLY. As shown in Fig. 3E, ACLY stability was compromised in SEC63-depleted cells. Overexpressed SEC63 elevated the stability of ACLY while SEC63 T537A mutant diminished this effect (Fig. 3F).

The ubiquitin–proteasome system (UPS) is important for controlling ACLY stability [26]. Thus, the proteasome inhibitor, MG132, was used for determining if SEC63-mediated ACLY stabilization depends on UPS. ACLY reduction by SEC63 depletion was reversed by the addition of MG132, indicating that SEC63 regulates ACLY levels by proteasome-dependent pathway (Fig. 3G). The ubiquitination of ACLY in HCC cells was decreased under ER stress while the effect was almost invisible when SEC63 was knocked down (Fig. 3H). Furthermore, the ubiquitination of ACLY was upregulated when SEC63 was depleted (Fig. 3I). Consistently, overexpressed SEC63 inhibited ubiquitination of ACLY while T537A mutation of SEC63 diminished this effect (Fig. 3J). These results demonstrate that SEC63 increases the stability of ACLY by inhibiting ACLY ubiquitination. Next, we uncovered the mechanism by which SEC63 affects ubiquitination of ACLY. Cullin 3 (CUL3) interacts with ACLY through its adaptor protein, KLHL25 (Kelch-like family member 25), for ubiquitinating and degrading ACLY [33]. Intriguingly, SEC63 binds to the fragment A3 (amino acids 621–820) (Fig. 1J) which also interacts with KLHL25, indicating that SEC63 might compete with KLHL25 for binding to ACLY [26]. Indeed, Flag-SEC63 inhibited the interaction between KLHL25 and ACLY and this effect was reinforced by phosphorylation of SEC63 (Fig. 3K). Moreover, we observed the decreased interaction between KLHL25 and ACLY after ER stress in control cells rather than SEC63-depleted cells (Fig. 3L). The interaction between SEC63 and ACLY was enhanced by ER stress in both nucleus and cytoplasm (Supplementary Fig. S3B). Consistently, ER stress failed to induce ACLY stabilization in SEC63-depleted cells (Fig. 1L). Together, these findings demonstrate that SEC63 improves the stability of ACLY by counteracting the interaction between KLHL25 and ACLY.

### Upregulation of SEC63 correlates with poor prognosis of HCC patients

**Fig. 4:**
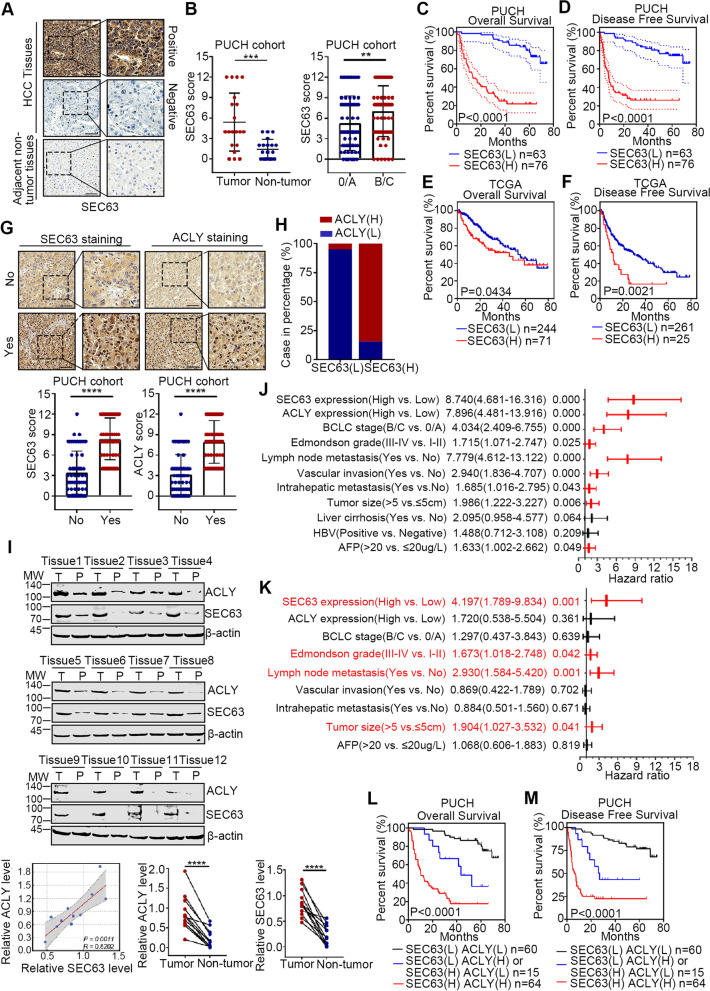
SEC63 is associated with prognosis of HCC patients. **A** Representative immunohistochemistry staining of SEC63 in 139 cases of HCC tissues and 20 cases of non-tumorous tissues. Scale bars, 50 μm. **B** Summary of SEC63 expression in HCC tissues and their non-tumorous counterparts. **C-D** Kaplan–Meier survival curve for OS (C) and DFS (D) of the HCC patients with different level of SEC63. **E–F** The OS and DFS curve of the HCC patients with different SEC63 mRNA levels from TCGA-LIHC were analyzed by Kaplan–Meier analyses. **G** Representative immunostaining of SEC63 in HCC tissues with metastasis (Yes) or without metastasis (No). Staining scores of SEC63 and ACLY in HCC tissues with (*n* = 76) or without metastasis (*n* = 63) were showed in histograms. **H** The correlation between SEC63 staining and ACLY staining in the HCC tissues was analyzed. **I** Western blot analysis of SEC63 and ACLY expression in 12 individual paired HCC tissues. **J** Univariate analyses of prognostic factors in terms of OS of HCC patients. **K** Multivariate analyses of prognostic factors in terms of OS of HCC patients. **L-M** OS **(L)** and DFS **(M)** curves of HCC patients were plotted against the SEC63 and/or ACLY expression levels using the Kaplan–Meier method. The difference between the survival curves was analyzed using the log-rank test

Cancer cells own the ability to hijack ER stress pathways to support their malignant activities [4]. Considering that SEC63 is activated to upregulate ACLY by ER stress, we next analyzed the clinical relevance of SEC63 with HCC. We first detected SEC63 expression in 139 cases of HCC specimens (Fig. 4A). SEC63 was upregulated in HCC tissues and increased at clinical stage B/C (Fig. 4B). Moreover, higher mRNA expression levels of SEC63 were also observed in HCC patients from TCGA-LIHC, ICGC-LIRI-JP and GSE124535 cohorts (Supplementary Fig. S4A). High SEC63 expression in HCC patients was associated with shorter OS and DFS time (Fig. 4C-D). Consistently, data analysis from TCGA-LIHC cohort also indicated that SEC63 high expression predicted poor clinical outcomes than those with SEC63 low expression (Fig. 4E-F). The similar results were obtained using data from ICGC-LIRI-JP cohort (Supplementary Fig. S4B). Given that SEC63 was higher in late-stage (B/C) HCC compared to early-stage (0/A), we explored the relation between SEC63 or ACLY expression and HCC metastasis and found that increased expression of SEC63 and ACLY in metastatic HCC tissues compared with non-metastatic ones (Fig. 4G). Taken together, these results suggest that increased SEC63 and ACLY expression levels are associated with HCC metastasis and can be used as potential indicators of poor prognosis of HCC.

Moreover, ACLY was upregulated in SEC63-overexpressed HCC tissues compared with SEC63 low expression group (Fig. 4H). The expression of SEC63 and ACLY on 12 paired cancerous and matched noncancerous samples of human HCC tissues was further detected by immunoblotting (Fig. 4I). Similarly, SEC63 expression was significantly correlated with ACLY in HCC tissues. Importantly, univariate and multivariate Cox regression analyses showed that besides Edmondson Grade, metastasis, and tumor size, the expression of SEC63 was also identified as an independent prognostic indicator for prognosis of HCC patients (Fig. 4J-K). Finally, the survival analysis showed that the OS of patients with co-upregulation of SEC63 and ACLY expression was shortest than that of other patients (Fig. 4L). Co-upregulation of SEC63 and ACLY expression also predicted poorest DFS compared to patients with low SEC63 and/or low ACLY expression (Fig. 4M). Data analysis from TCGA-LIHC and ICGC-LIRI-JP cohorts suggested similar phenomena (Supplementary Fig. S4C-D). These findings indicate that co-upregulation of SEC63 and ACLY plays important roles in HCC progression.

### SEC63 promotes HCC cell metastasis relying on ACLY

**Fig. 5:**
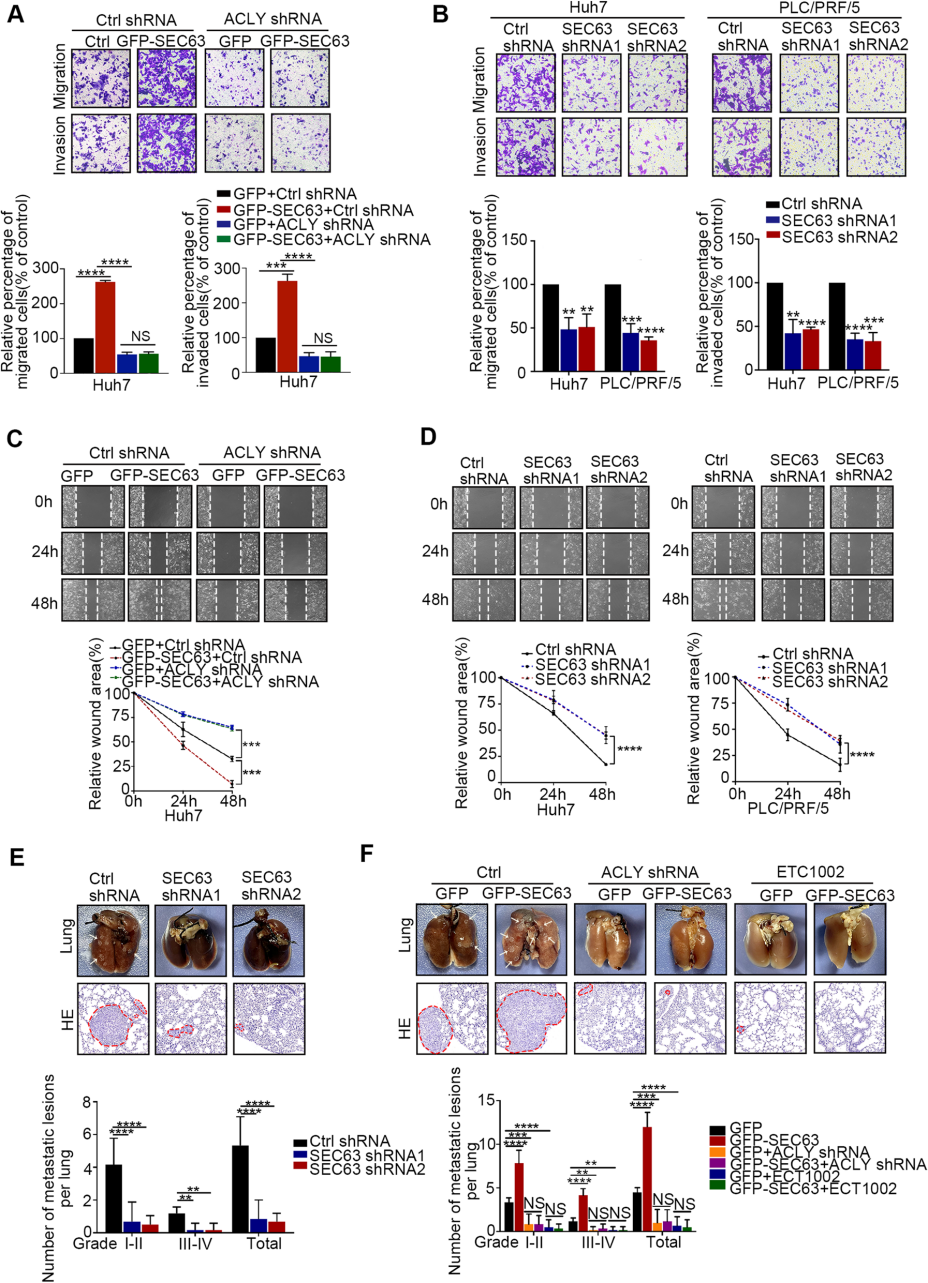
SEC63 promotes HCC cell metastasis relying on ACLY. **A** Migration assay or invasion assay was performed as indicated. Migration or invasion rate was further quantified (*n* = 3). **B** Migration assay or invasion assay was performed in SEC63-depleted HCC cells. Migration or invasion rate was further quantified (*n* = 3). **C** Wound healing assay was performed as indicated. Wound closure rate was quantified (*n* = 3). **D** Wound healing assay was performed in SEC63-depleted or Ctrl HCC cells. Wound closure rate was quantified (*n* = 3). **E** Huh7 cells without (shCtrl) or with SEC63 knockdown (shSEC63) were injected into BALB/c nude mice to establish a lung metastasis model (*n* = 6). **F** Huh7 cells were injected as indicated into BALB/c nude mice to establish a lung metastasis model. Moreover, the mice were treated with vehicle or ETC1002 (30 mg/kg) per 2 days for 8 weeks (*n* = 6). The data are expressed as the mean ± SD

Notably, upregulation of SEC63 is associated with HCC metastasis. The prognosis of HCC is very dismal due to the high rate of metastasis [19]. We thus determined functional roles of SEC63 in HCC cell metastasis. The endogenous SEC63 was stably knocked down and ectopic SEC63 was stably overexpressed in HCC cells (Supplementary Fig. S5A-B). We firstly analyzed the effect of SEC63 on cell proliferation. The 3-(4,5-dimethylthiazol-2-yl)-2,5-diphenyltetrazolium bromide (MTT) assays were conducted to show that SEC63 had a small impact on HCC cell proliferation (Supplementary Fig. S5C-F). Importantly, overexpression of SEC63 resulted in a dramatic increase of cell migration and invasion of HepG2 and Huh7 cells (Fig. 5A and Supplementary Fig. S5G). These phenomena were not observed in ACLY-depleted cells. Similarly, SEC63 knockdown reduced the migration and invasion abilities of HCC cells (Fig. 5B). Wound healing assay further confirmed that overexpression of SEC63 promoted migration of HCC cells in ACLY-expressed cells (Fig. 5C and Supplementary Fig. S5H). Similarly, SEC63 knockdown reduced the migration abilities of HCC cells (Fig. 5D). To verify these results, we further explored the effects of SEC63 knockdown or overexpression on in vivo metastasis of HCC xenografts by intravenous injection of the cells into nude mice. As shown in Fig. 5E, loss of SEC63 displayed to decrease the formation of lung metastases. Consistently, increased SEC63 expression promoted the formation of lung metastases (Fig. 5F). Moreover, SEC63-mediated metastasis was mainly inhibited by ACLY knockdown or ACLY inhibitor (ECT1002) (Fig. 5F). Accordingly, the expression of SEC63 was positively correlated with Snail1 level (Supplementary Fig. S5I). Together, our results demonstrate that SEC63 enhances HCC metastasis by regulating ACLY and targeting SEC63 shows the potential in attenuating HCC progression.

### SEC63 induces epithelial-mesenchymal transition (EMT) in HCC cells by upregulating Snail1 expression

**Fig. 6:**
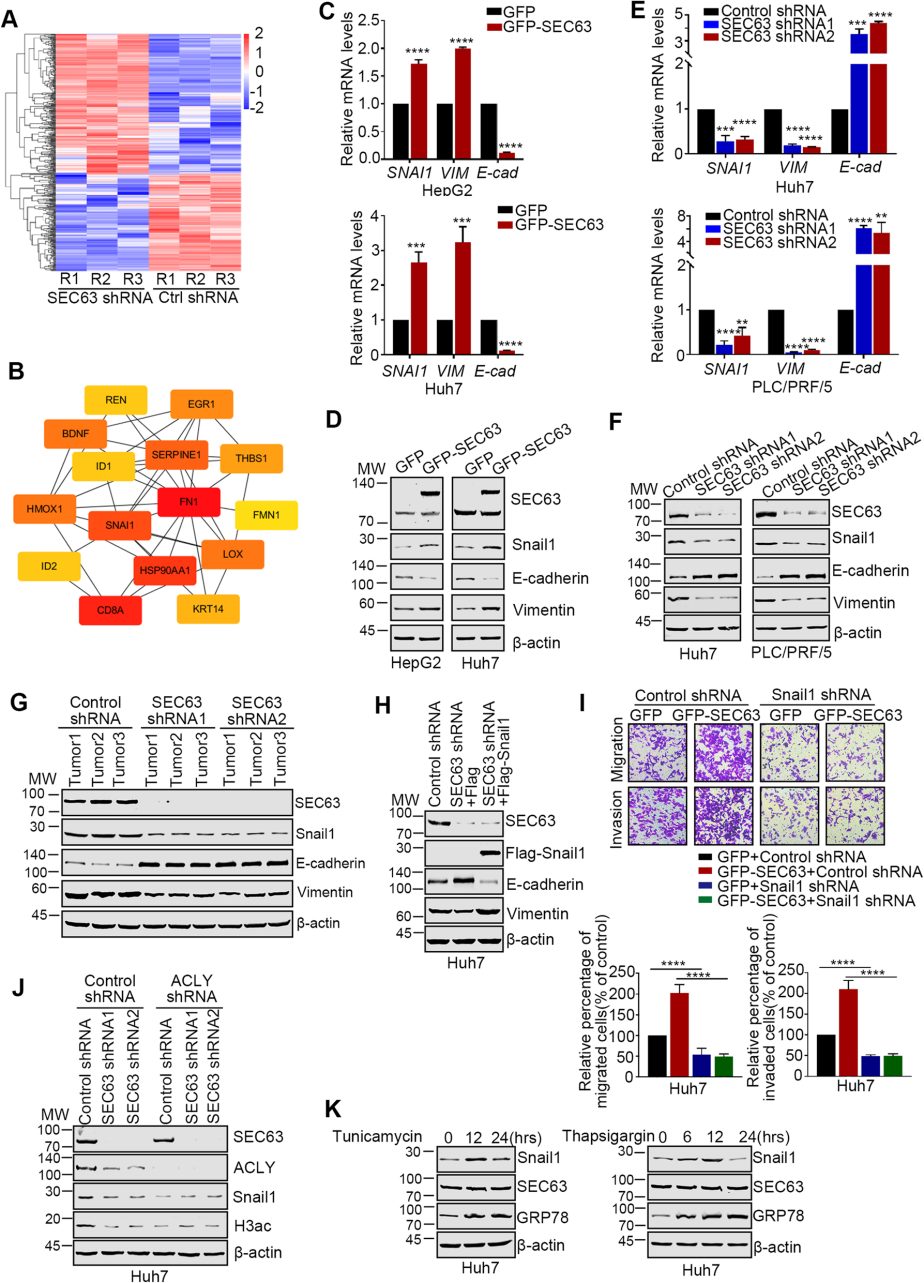
SEC63 regulates the expression of Snail1 in HCC cells. **A** Heatmap shows differentially expressed genes (DEGs) upon SEC63 knockdown in Huh7 cells. Upregulated and downregulated genes are colored in red and blue, respectively. **B** Protein–protein interaction analysis reveals the hub genes among DEGs by STRING. **C** Total RNAs were extracted from SEC63-overexpressed or control cells. Then, RT-qPCR was performed as indicated. *n* = 3. **D** Cell lysates from HepG2 or Huh7 cells were subjected to western blot. **E** Total RNAs were extracted from SEC63-depleted or control cells. Then, RT-qPCR was performed. *n* = 3. **F** Cell lysates from SEC63 knockdown or control cells were subjected to western blot using the indicated antibodies. **G** Western blot analysis of the indicated proteins in the mice xenograft tumor tissues. **H** Huh7 cells were transfected as indicated. Western blot was performed for the indicated proteins. **I** Migration assay or invasion assay was performed as indicated. *n* = 3. **J** Cell lysates from Huh7 cells were subjected to western blot for evaluating the expression level of the indicated proteins. **K** Huh7 cells were treated with TM (5 μg/mL) or TG (1 μM) for the indicated time points. Western blot was performed using the anti-Snail1, anti-ACLY or anti-GRP78 antibodies. The data are expressed as the mean ± SD

To clarify the mechanism by which SEC63 promotes HCC metastasis, we carried out RNA sequencing using purified RNAs from SEC63-depleted and control cells. Totally, 433 genes were identified to be differentially expressed (Fig. 6A). Protein–protein interaction analysis by STRING revealed that the top 15 hub genes were mainly involved in cell migration and invasion (Fig. 6B). Among these genes, Snail1 (coded by *SNAI1*) is an important transcriptional factor in EMT, which is a well-accepted biological process in tumor cell metastasis [34, 35]. We thus examined the effect of SEC63 modulation on the expression of Snail1 and its downstream targets in HCC cells. In SEC63-overexpressed HCC cells, the mRNA levels of Snail1 and Vimentin (two of mesenchymal markers) were significantly increased, while the mRNA level of E-cadherin (the epithelial marker) was reduced in HCC cells (Fig. 6C). Western blot also revealed the similar effects (Fig. 6D). Moreover, depletion of SEC63 decreased the expression of Snail1 and Vimentin while increased the expression of E-cadherin (Fig. 6E). Western blot also showed that SEC63 depletion caused a decreased expression of Snail1 (Fig. 6F). Consistently, SEC63 level was significantly associated with Snail1 in xenograft tissues (Fig. 6G). These data suggest that SEC63 regulates Snail1 expression to promote EMT of HCC cells.

To analyze whether SEC63 induces HCC cell metastasis through downregulating Snail1 expression, we re-introduced Snail1 in SEC63-depleted cells. As shown Fig. 6H, reintroduction of Snail1 expression significantly reversed the reduced EMT phenotype of SEC63 depletion back to similar levels of control cells. Furthermore, the overexpression of SEC63 had no impact on HCC cell migration and invasion in Snail1 repressed cell (Fig. 6I). It’s of interest whether ACLY is required for SEC63-upregulated Snail1 expression. We found that ACLY depletion dramatically attenuated the effect of SEC63 on the expression level of Snail1 and acetylation level of histone H3 (Fig. 6J). On the other hand, the Snail1 level was also increased under ER stress (Fig. 6K). Altogether, SEC63 upregulates Snail1 expression to enhance HCC metastasis by stabilizing ACLY, which is reinforced upon ER stress.

### SEC63 promotes ACLY-related metabolic reprogramming

**Fig. 7:**
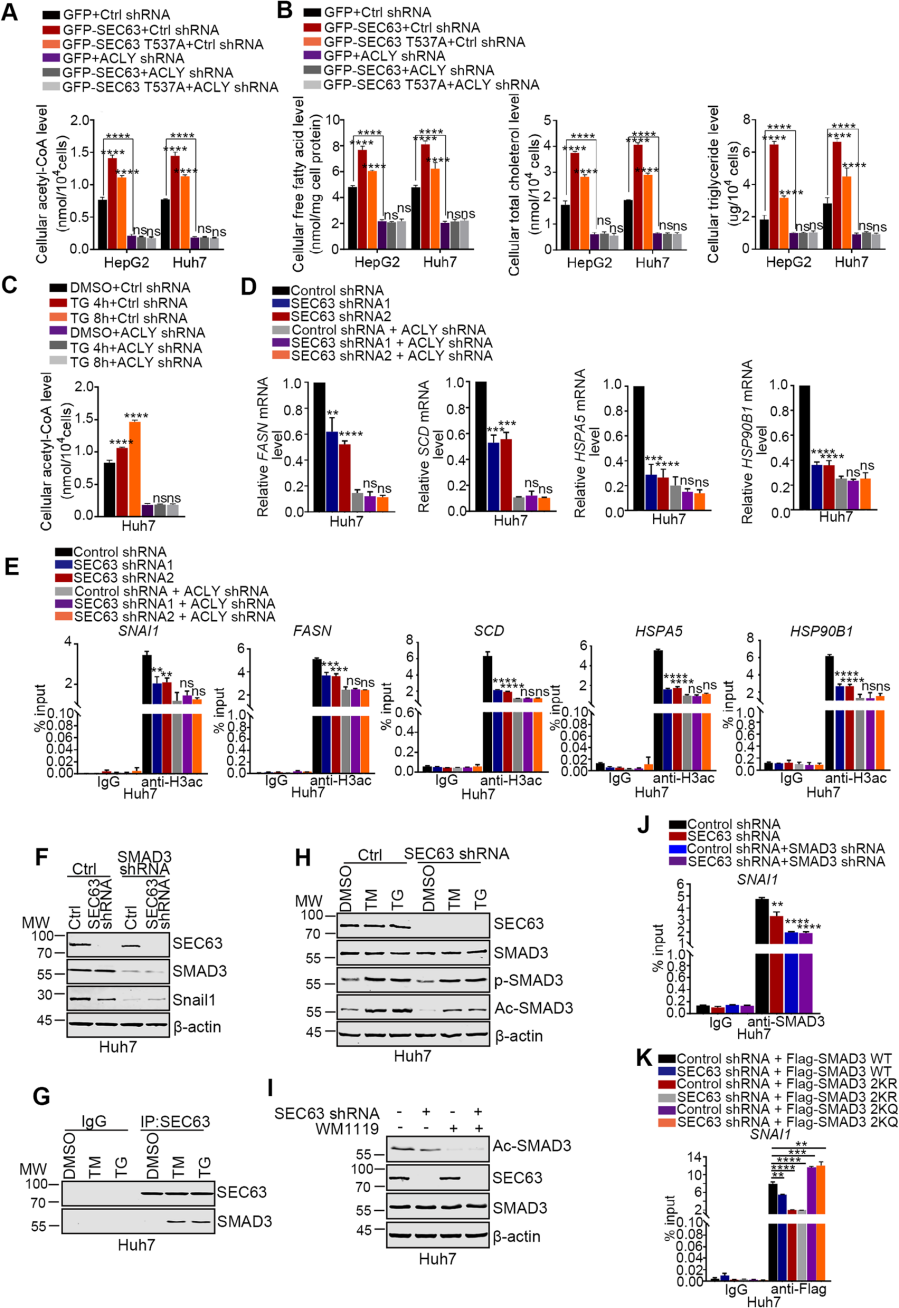
SEC63 modulated ACLY-related metabolic and epigenetic reprogramming. **A** The cellular acetyl-CoA level was evaluated in the HepG2 and Huh7 cells (*n* = 4). **B** The cellular free fatty acids, cholesterol, and triglyceride levels were evaluated in the HepG2 and Huh7 cells (*n* = 4). **C** The cellular acetyl-CoA level was detected in the Huh7 cells transfected as indicated (*n* = 4). **D** Total RNAs were extracted from the Huh7-ctrl or Huh7-shSEC63 cells transfected with ACLY shRNA. RT-qPCR was performed for the UPR target genes (*FASN*, *SCD*, *HSPA5*, *HSP90B1*) (*n* = 3). **E** ChIP-qPCR was performed using anti-acetyl-H3 antibodies (*n* = 3). **F** Western blot analysis of indicated protein levels in Huh7 cells. **G** The cells were treated with TM (5 μg/mL) or TG (1 μM) for 8 h and immunoprecipitation was performed with anti-SEC63 antibody. **H** The Huh7-ctrl or Huh7-shSEC63 cells were treated with TM or TG. Western blot was further performed using the indicated antibodies. **I** The ctrl cells or SEC63-depleted cells were treated with WM1119 (KAT6A inhibitor). Western blot was performed for the indicated proteins. **J, K** ChIP assays were performed with IgG, anti-SMAD3 or anti-Flag antibody followed by qPCR to amplify the *SNAI1* promoter region. The data are shown as the mean ± SD

ACLY is a critical enzyme catalyzing the conversion of citrate to oxaloacetate and acetyl-CoA, which is the building block for biosynthesizing lipids, including free fatty acids, cholesterol, and phospholipids [36]. Upon ER stress, IREα pathway increases lipid biosynthesis to support ER membrane [37]. Moreover, ACLY-mediated regulation of acetyl-CoA production is also crucial for tumor metabolic reprogramming [38]. Thus, it is of great importance to figure out whether SEC63 modulates ACLY-related metabolic reprogramming. As expected, SEC63 knockdown caused corresponding decreases in the acetyl-CoA level, accompanied by reduced total cholesterol, triglycerides and free fatty acid levels (Supplementary Fig. S6A-B). In lines with this, overexpression of SEC63 increased cellular acetyl-CoA level as well as lipids levels (Fig. 7A-B). SEC63 T537A mutation attenuated this effect. Moreover, SEC63 had little impact on acetyl-CoA and lipids levels in the absence of ACLY in HCC cells. Additionally, the acetyl-CoA level was increased after ER stress in control cells but not in ACLY-suppressed cells (Fig. 7C). Therefore, these findings indicate that SEC63 enhances ACLY-mediated acetyl-CoA production, thereby increasing lipogenesis, to support ER membrane upon ER stress.

### SEC63 alters epigenetic reprogramming to enhance UPR

The cellular acetyl-CoA levels play important roles in the sustenance of histone acetylation [10]. To further delineate the effect of SEC63-mediated ACLY stabilization, we analyzed histone acetylation levels after SEC63 depletion. A significant decline in global acetylated levels of histones H3 was observed after SEC63 depletion. Moreover, this effect was not found after SEC63 ablation in ACLY-depleted cells (Fig. 6J). Thus, these findings indicate that upregulation of ACLY by SEC63 promotes acetylation of H3 through increasing cellular acetyl-CoA level.

ACLY-related histone acetylation participates in modulating the transcription of downstream genes. After ER stress, IRE1α is activated to induce the expression of XBP1s, which is an active transcription factor. XBP1s upregulates the expression of UPR target genes, such as ER chaperones (*HSPA5*, *HSP90B1*, and *CALR*) and lipid metabolism (*SCD1*, *DGAT2*, and *FASN*) [39]. It’s unknown whether ACLY is involved in modulating UPR target genes. To address this issue, we analyzed the effect of SEC63 and ACLY on UPR target genes. As shown in Fig. 7D, depletion of SEC63 significantly reduced the expression of the target genes under ER stress conditions. Moreover, knockdown of ACLY abrogated this effect. Concurringly, H3 acetylation was decreased at the promoter region of these genes after SEC63 knockdown and this effect was abolished when ACLY was silenced (Fig. 7E). Together, these findings reveal that upregulation of ACLY by SEC63 specifically offers sufficient acetyl-CoA to alter the epigenetic profile for supporting UPR.

Intriguingly, we observed decreased H3 acetylation levels at the promoter region of *SNAI1* in SEC63 depleted cells compared to those in control cells (Fig. 7E), which is consistent with reduced expression of Snail1 in SEC63 depleted cells (Fig. 6E). These results indicate that SEC63 epigenetically modulates expression of Snail1. To elucidate the underlying mechanism, we identified the SEC63-interacting protein upon ER stress. Pathway analysis revealed that SEC63 interacting proteins were mainly enriched in metabolic pathway, protein processing in ER, and adhesion-related proteins. Among the adhesion-related protein, SMAD3 is of interest to us due to its highest scores in mass spectrometry (Supplementary Fig. S6C). Moreover, it has been found that SMAD3 promotes Snail1 transcription [40]. SEC63 failed to regulate Snail1 expression in SMAD3-depleted cells (Fig. 7F and Supplementary Fig. S6D), indicating that SEC63 upregulates Snail1 in SMAD3-dependent way. The acetylation of SMAD3 at K20 and K117 in nucleus is critical for enhancing SMAD3 mediated transcriptional activity [41]. We hypothesized that SEC63 contributed to increase the acetylation of SMAD3 by stabilizing ACLY to offer acetyl-CoA. To test this possibility, we confirmed the binding between SEC63 and SMAD3 and the interaction was enhanced upon ER stress (Fig. 7G). Moreover, the acetylation of SMAD3 was dramatically increased upon ER stress and SEC63 knockdown decreased the acetylation of SMAD3 (Fig. 7H), implying the importance of SEC63 on acetylation of SMAD3 under ER stress. Previous studies show that IRE1α/STAT3 signaling increases phosphorylation of SMAD3 after ER stress [42, 43], which is indispensable for nuclear translocation of SMAD3 [44]. SEC63 had little effect on phosphorylation of SMAD3, suggesting that SEC63 mainly regulates SMAD3 in nucleus. Since lysine acetyltransferase 6A (KAT6A) is the critical acetyltransferase for SMAD3 [41], we tested the roles of SEC63 or ACLY knockdown on the acetylation of SMAD3 when cells were treated with KAT6A inhibitor (WM1119). The results revealed that the knockdown of SEC63 or ACLY had no impact on the SMAD3 acetylation level after KAT6A was inhibited (Fig. 7I and Supplementary Fig. S6E). Thus, SEC63 contributes to increase the acetylation of SMAD3 by binding to SMAD3 and stabilizing ACLY to offer acetyl-CoA. Furthermore, SEC63 knockdown reduced the interaction of SMAD3 with the promoter of *SNAI1* (Fig. 7J). After the construction of acetylation-defective SMAD3 2KR (K20R and K117R, mutation lysine to arginine) mutant and acetylation-mimic SMAD3 2KQ (K20Q and K117Q, mutation lysine to glutamine) mutant, we observed 2KR mutant decreased the binding of SMAD3 to the promoter of *SNAI1* and 2KQ increased the binding. Importantly, SEC63 only affected the binding of wild-type SMAD3 to the promoter of *SNAI1* (Fig. 7K), suggesting that SEC63-associated acetylation of SMAD3 modulates SMAD3-mediated transcription of *SNAI1*. Together, these data suggest that SEC63-associated acetylation of SMAD3 epigenetically promotes the expression of Snail1 to facilitate HCC metastasis in the nucleus upon ER stress.

Next, we analyzed the relations of SEC63, ACLY and SMAD3 in HCC tissues. The expression of SEC63 is positively associated with ACLY or SMAD3 expression (Supplementary Fig. S6F). Co-upregulation of SEC63/ACLY/SMAD3 expression predicted poorer prognosis compared to the rest patients in TCGA-LIHC cohort (Supplementary Fig S6G). Data analysis from ICGC-LIRI-JP cohort also suggested similar phenomena (Supplementary Fig. S6H). These findings indicate that the metastasis caused by SEC63/ACLY/SMAD3 interactions in the nucleus is critical for cancer cell adaption to ER stress as well as contributes to the poor outcome of HCC patients.

## Discussion

**Fig. 8:**
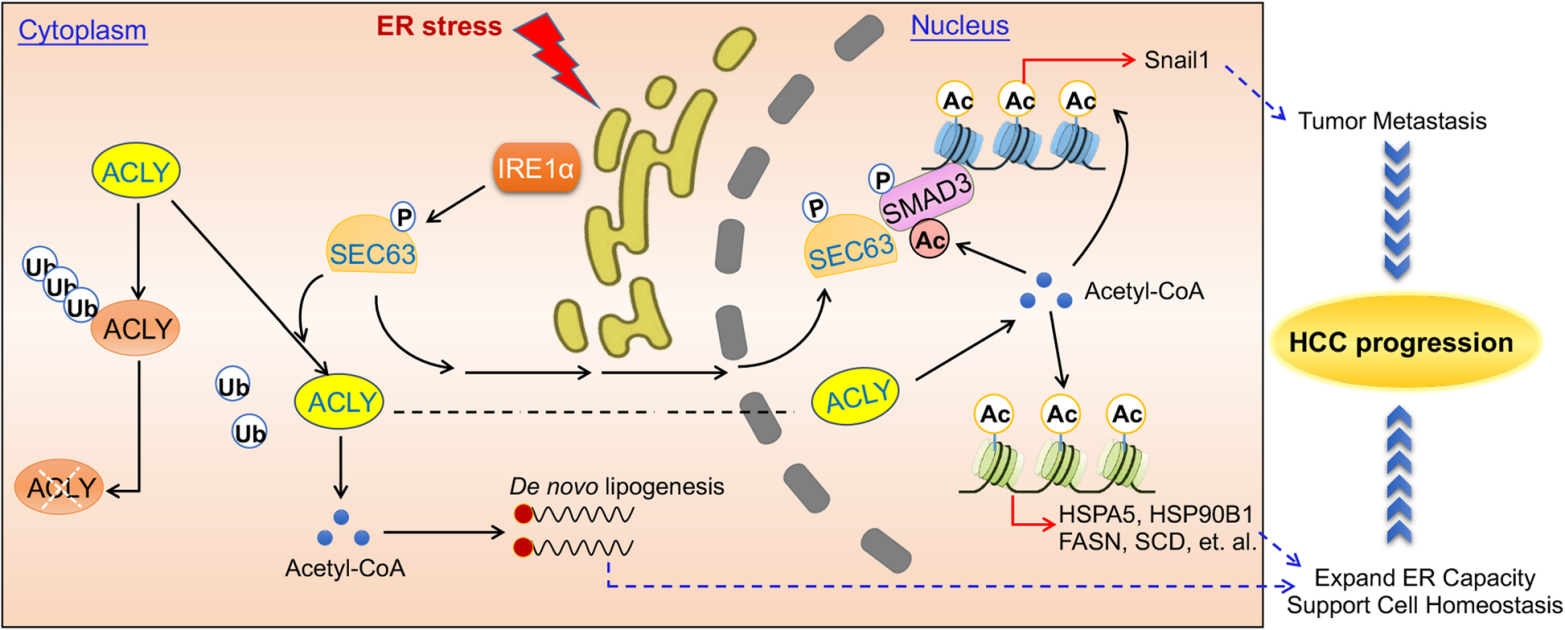
A working model explaining how SEC63 promotes metabolic reprogramming and epigenetic remodeling upon ER stress. Upon ER stress, SEC63 is phosphorylated at T537 by IRE1α. Phosphorylated SEC63 subsequently upregulates ACLY stability to produce more acetyl-CoA. Elevated acetyl-CoA is utilized for lipogenesis and histone acetylation for the transcription of UPR target genes. Meanwhile, SEC63 also enters into the nucleus together with ACLY. In the nucleus, SEC63 contributes to activation of UPR targets and induces Snail1 expression by increasing acetylation of SMAD3 to promote cancer cell metastasis. Upregulation of UPR targets and lipogenesis are beneficial for maintaining ER homeostasis to support cancer cell survival in response to stress. Metastasis is crucial for helping cancer cells seek new settlements upon microenvironmental stimuli. Abbreviations: ER, endoplasmic reticulum; HCC, hepatocellular carcinoma

Dysregulation of ER homeostasis is associated with human disorders including cancers [45]. Tumor cells are often exposed to microenvironmental stimuli which lead to ER stress [46, 47]. It’s observed that tumor cells acquire the ability to reprogram ER stress-related pathway to support their malignant activities [4, 45]. The underlying mechanisms have not yet been investigated. Here, we reported that cancer cells reprogram acetyl-CoA metabolic pathway in response to ER stress (Fig. 8).

Acetyl-CoA sustains lipid biosynthesis which is upregulated upon ER stress to maintain ER homeostasis [36]. ACLY plays an important role in Acetyl-CoA production [36]. ACLY inhibitor triggers ER stress, indicating that the enzymatic activity of ACLY is indispensable for ER functions [48]. It’s meaningful to determine how ACLY is regulated after ER stress. Here, we found that ACLY is stabilized to increase lipid synthesis in response to ER stress. Meanwhile, upregulated ACLY is also critical for the epigenetic activation of UPR target genes. The stability of ACLY is tightly controlled by post-translational modifications, including ubiquitination, phosphorylation, and acetylation [36]. CUL3, as a critical ubiquitin ligase, binds to ACLY through its adaptor protein, KLHL25, to ubiquitinate and degrade ACLY [33]. USP30 deubiquitinates ACLY to improve ACLY stability [15]. We identified SEC63 as the essential factor for stabilization of ACLY upon ER stress. SEC63 competes with KLHL25 to interact with ACLY, thereby inhibiting ubiquitination and degradation of ACLY. These findings uncover the importance of ACLY in response to ER stress.

The cellular acetyl-CoA is involved in cancer progression as a signal for cell growth or metastasis [13, 16]. As one of key enzyme in Acetyl-CoA biosynthesis, ACLY is associated with various malignancies, such as colorectal, gastric, and cervical cancers [49]. Overexpression of ACLY correlates with poor outcomes of human cancers such as lung adenocarcinoma and acute myeloid leukemia [26, 50]. We also observed that ACLY is overexpressed in HCC tissues and high ACLY level predicts poor prognosis of HCC patients. The severe or prolonged ER stress often causes cell death because of dysfunction of ER [6]. Interestingly, cancer cells show the larger capacity to severity of the stimuli and can hijack ER stress pathways for supporting the malignant activities [4]. Our findings indicated that the upregulation of ACLY is involved in protecting tumor cells from ER stress. Thus, ACLY appears as a potential target for cancer therapy. The inhibitors of ACLY, including SB-204990 and ETC1002, have shown the latent anti-tumor effects [26, 51]. We observed that ETC1002 attenuate HCC metastasis in mice xenografts. It will be meaningful to explore whether ACLY inhibitor could be applied for HCC treatment in the future study.

The transport of immature proteins into and across the ER membrane is a highly conserved process and is indispensable for the synthesis of transmembrane and secretory proteins in eukaryotic cells [27]. The protein transport machinery as core element for this transport includes the ER proteins SEC61, SEC62 and SEC63 [52–54]. SEC63 interacts with chaperones to assist the translocation of precursor proteins [21, 54, 55]. These studies identified the importance of SEC63 in the transport of precursor proteins. During prolonged ER stress, SEC63 recruits and activates Bip ATPase to bind onto IRE1α to inhibit oligomerization of IRE1α and attenuate excess IRE1α RNase activity [25]. Interestingly, SEC63 also locates in cytoplasm and nucleus besides ER. It is important to uncover the functions of non-ER-resident SEC63. We found that SEC63 binds to ACLY in cytoplasm and nucleus and protects ACLY from proteasome-dependent degradation in the early stage of ER stress. SEC63 mediated stabilization of ACLY contributes to increase lipogenesis and enhance the expression of UPR target genes, thereby improving ER homeostasis upon primitive stress. Our findings further highlight the critical roles of SEC63 in keeping ER homeostasis. It is unknown how SEC63 is regulated. Previous studies reported that SEC63 is phosphorylated by CK2 at serine 574, serine 576 and serine 748 and these phosphorylation sites enhance the interaction between SEC63 and SEC61 to form the protein transport machinery [56]. Our findings identified SEC63 as a novel downstream target of IRE1α. IRE1α phosphorylates SEC63 at T537 upon ER stress. Phosphorylation of SEC63 at T537 is helpful to aggravate the relations between SEC63 and ACLY and is critical for increasing nuclear SEC63 level. Thus, these findings revealed that SEC63 is precisely controlled by posttranslational modification. However, SEC63 T537A mutant only partially restored the ubiquitination level of ACLY. It’s possible that other phosphorylation sites or posttranslational modifications, such as acetylation and ubiquitination, are involved in regulating functions of SEC63. This needs to be explored in the future study.

Mutations in the gene encoding SEC63 are tightly associated with polycystic liver disease [57]. Subsequent studies confirmed *SEC63* as a driver gene in the pathogenesis of polycystic liver disease [57, 58]. Importantly, amplification or overexpression of SEC63 was reported to be frequent molecular characteristics of human cancers [59]. Frameshift mutations in *SEC63* gene caused by microsatellite instability were uncovered in microsatellite-unstable cancers [60]. These studies indicate a potential role of SEC63 in cancer development and progression. We found that SEC63 is upregulated in human HCC tissues. Importantly, overexpression of SEC63 predicts poor prognosis of HCC patients and co-upregulation of SEC63 and ACLY predicts poorer prognosis. Moreover, the mRNA expression levels of SEC63 and ACLY are correlated in HCC tissues. The depletion of SEC63 had no effect on the mRNA level of ACLY in HCC cells, indicating that SEC63 does not affect the mRNA level of ACLY. It’s possible that the mRNAs of ACLY and SEC63 are regulated by the same upstream factors, such as microRNAs, transcriptional factors or RNA modifications. However, the underlying mechanism has not yet been studied. It is worth for further study. Our data suggest that SEC63 promotes HCC metastasis relying on ACLY. Mechanistically, nuclear SEC63 increases the acetylation of SMAD3 to induce the expression of Snail1 in an ACLY-dependent manner. We observed a significant reduction of lung metastasis by SEC63 deficiency in an in vivo model. The induction of lung metastasis by SEC63 overexpression was also demonstrated, which can be reversed by ACLY inhibitor. Metastasis caused by SEC63 is beneficial for cancer cells to seek new settlements upon ER stress caused by the primary microenvironment. Thus, our data indicate that IRE1α-SEC63-ACLY-Snail1 axis is critical to drive HCC metastasis. It is a potential therapeutic target for HCC metastasis.

## Conclusions

In summary, we reported the important roles of IRE1α-SEC63-ACLY signaling in regulating HCC metabolism and metastasis. After ER stress, SEC63 is phosphorylated at T537 by IRE1α pathway. Phosphorylated SEC63 subsequently upregulates ACLY stability to produce more acetyl-CoA. Elevated acetyl-CoA is utilized for lipogenesis and histone acetylation for the transcription of UPR target genes. At the same time, SEC63 induces Snail1 expression by increasing acetylation of SMAD3 to promote cancer cell metastasis. These findings highlight a cancer selective adaption, whereby HCC cells deploy ER stress-related responses to keep cell survival and initiate cell metastasis.

## Supplementary Information

**Additional file 1: Figure S1.** ACLY is upregulated by SEC63 in response to ER stress in HCC cells. A The heatmaps displaying the differentially expressed ARGs from TCGA-LIHC, ICGC-LIRI-JP, and GSE101728 cohorts. B The expression level of the indicated genes was analyzed in the indicated cohorts. C The overall survival curve of the HCC patients with different ACLY mRNA levels from ICGC was analyzed by Kaplan-Meier analyses. D Triple immunofluorescene (IF) staining for ACLY (red), SEC63 (green), and nuclei (DAPI, blue) was performed in Huh7 and HepG2 cells. Scale bar, 10 μm. E GST pulldown assay with purified fragments as indicated. **Figure S2.** SEC63 is phosphorylated at T537 after ER stress. A Western blot analysis of SEC63 in cell lysates of Huh7 cells treated as indicated. B Relative SEC63 expression levels in HepG2 and Huh7 cells were determined by immunofluorescence assay using Leica LAS X confocal software (n = 100). C The predication analysis of SEC63 localization by COMPARTMENTS. D Cell fraction was performed and western blot was employed for analyzing the indicated protein. E Dot blot analysis was performed for validating T537-p antibody. F Cell lysates from Huh7 cells were subjected to Co-IP using the indicated antibody. G Cell fraction was performed and the indicated proteins were determined by western blot. **Figure S3.** SEC63 increases the stability of ACLY. A Total RNA extracted from the Huh7 cells treated with TM (5 μg/mL) or TG (1 μM) were subjected to RT-qPCR as indicated. B HepG2 and Huh7 cells were treated with TM (5 μg/mL) or TG (1 μM) and immunofluorescence was performed. Scale bar, 10 μm. **Figure S4.** SEC63 is associated with prognosis of HCC patients. A The mRNA level of SEC63 was analyzed as indicated. B The overall survival curve of the HCC patients with different SEC63 mRNA levels from ICGC database was analyzed by Kaplan-Meier analyses. C The overall survival and disease-free survival curves of HCC patients were plotted against the SEC63 and/or ACLY mRNA levels based on the prognosis data of TCGA-LIHC database using the Kaplan-Meier method. The difference between the survival curves was analyzed using the log-rank test. D Overall survival curve of HCC patients was plotted against the SEC63 and/or ACLY mRNA levels based on the prognosis data of ICGC database using the Kaplan-Meier method. The difference between the survival curves was analyzed using the log-rank test. **Figure S5. **SEC63 promotes HCC cell metastasis relying on ACLY. A HepG2 or Huh7 stable cell lines with overexpression of SEC63 were generated. Western blot was performed for analyzing the expression level of SEC63. B Huh7 or PLC/PRF/5 stable cell lines with knockdown of SEC63 were generated. The knockdown effect was determined by western blot. C-F The cells were treated as indicated and cell proliferation was assessed by MTT assay. G Migration assay or invasion assay was performed as indicated. Migration or invasion rate was further quantified (n = 3). H Wound healing assay was performed as indicated. Wound closure rate was quantified (n = 3). I Western blot analysis of the indicated proteins in 12 individual paired HCC tissues used in Figure 4I. **Figure S6. **SEC63 modulates ACLY-related metabolic and epigenetic reprogramming. A The cellular acetyl-CoA level was evaluated in the indicated cells (n = 4). B The cellular free fatty acids, cholesterol level, and triglyceride levels were evaluated in the indicated cells (n = 4). C The potential interacting proteins of SEC63 related to ER stress were identified by liquid chromatography-tandem mass spectrometry. D Huh7 cells were transfected as indicated. Then, total RNA was extracted and RT-qPCR was performed using the *SNAI1 *primers (n = 3). E The ctrl cells or ACLY-depleted cells were treated with WM1119. Western blot was performed for the indicated proteins. F The correlation analyses were analyzed as indicated using TCGA HCC dataset. G The overall survival or disease-free survival curves were analyzed as indicated using TCGA HCC dataset. H The overall survival curve was plotted as indicated using ICGC dataset. **Table S1.** Correlation of SEC63/ACLY expression with clinicopathologic status in 139 cases of patients with HCC. **Table S2.** Reagents and antibodies. **Table S3.** shRNA sequence. **Table S4.** Primer used for RT-qPCR.

## Data Availability

All publicly available data can be acquired from the corresponding web servers described in the materials and methods. The data that support the findings of this study are available from the corresponding author upon reasonable request.

## Abbreviations

ER: Endoplasmic reticulum
HCC: Hepatocellular carcinoma
UPR: Unfolded protein response
PERK: Protein kinase RNA-like ER kinase
IRE1α: Inositol-requiring enzyme 1α
ATF6: Activating transcription factor 6
ACLY: Adenosine triphosphate (ATP)-citrate lyase
DLAT: Dihydrolipoamide S-acetyltransferase
CS: Citrate synthase
ACACB: Acetyl-CoA carboxylase beta
ACSS1: Acetyl-CoA synthetase 1
ACSS2: Acetyl-CoA synthetase 2
ARGs: Acetyl-CoA metabolism-related genes
HSPA5: Heat shock protein family A member 5
HSP90B1: Heat shock protein 90 beta family member 1
CALR: Calreticulin
SCD1: Stearoyl-CoA desaturase 1
DGAT2: Diacylglycerol O-acyltransferase 2
FASN: Fatty acid synthase
SNAI1: Snail family transcriptional repressor 1
IHC: Immunohistochemistry
IF: Immunofluorescence
TCGA: The Cancer Genome Atlas
ICGC: International cancer genome consortium
GEO: Gene expression omnibus
siRNA: Short interfering RNA
GST: Glutathione S-transferase
RT-qPCR: Quantitative real-time PCR
ChIP: Chromatin immunoprecipitation
PPI: Protein–protein interaction
STRING: Search tool for recurring instances of neighbouring genes
TG: Thapsigargin
TM: Tunicamycin
OS: Overall survival
DFS: Disease-free survival
Co-IP: Co-immunoprecipitation
NLS: Nuclear localization signal
CHX: Cycloheximide
UPS: Ubiquitin–proteasome system
CUL3: Cullin 3
KLHL25: Kelch-like family member 25
WT: Wild-type
delN: Deletion mutant
MTT: 3-(4,5-Dimethylthiazol-2-yl)-2,5-diphenyltetrazolium bromide
EMT: Epithelial-mesenchymal transition
PUCH: Peking University Cancer Hospital
BCLC: Barcelona Clinical Liver Cancer
