# Supplementary material for: Activation of ACLY by SEC63 deploys metabolic reprogramming to facilitate hepatocellular carcinoma metastasis upon endoplasmic reticulum stress
Source: J Exp Clin Cancer Res. 2023 May 1;42:108. doi: 10.1186/s13046-023-02656-7 (PMC10150531; doi:10.1186/s13046-023-02656-7)
Supplement: Supplementary file 1 — Additional file 1: Figure S1. ACLY is upregulated by SEC63 in response to ER stress in HCC cells. A The heatmaps displaying the differentially expressed ARGs from TCGA-LIHC, ICGC-LIRI-JP, and GSE101728 cohorts. B The expression level of the indicated genes was analyzed in the indicated cohorts. C The overall survival curve of the HCC patients with different ACLY mRNA levels from ICGC was analyzed by Kaplan-Meier analyses. D Triple immunofluorescene (IF) staining for ACLY (red), SEC63 (green), and nuclei (DAPI, blue) was performed in Huh7 and HepG2 cells. Scale bar, 10 μm. E GST pulldown assay with purified fragments as indicated. Figure S2. SEC63 is phosphorylated at T537 after ER stress. A Western blot analysis of SEC63 in cell lysates of Huh7 cells treated as indicated. B Relative SEC63 expression levels in HepG2 and Huh7 cells were determined by immunofluorescence assay using Leica LAS X confocal software (n = 100). C The predication analysis of SEC63 localization by COMPARTMENTS. D Cell fraction was performed and western blot was employed for analyzing the indicated protein. E Dot blot analysis was performed for validating T537-p antibody. F Cell lysates from Huh7 cells were subjected to Co-IP using the indicated antibody. G Cell fraction was performed and the indicated proteins were determined by western blot. Figure S3. SEC63 increases the stability of ACLY. A Total RNA extracted from the Huh7 cells treated with TM (5 μg/mL) or TG (1 μM) were subjected to RT-qPCR as indicated. B HepG2 and Huh7 cells were treated with TM (5 μg/mL) or TG (1 μM) and immunofluorescence was performed. Scale bar, 10 μm. Figure S4. SEC63 is associated with prognosis of HCC patients. A The mRNA level of SEC63 was analyzed as indicated. B The overall survival curve of the HCC patients with different SEC63 mRNA levels from ICGC database was analyzed by Kaplan-Meier analyses. C The overall survival and disease-free survival curves of HCC patients were plotted against the SEC63 and/or ACL [file 13046_2023_2656_MOESM1_ESM.pdf]

Supplementary materials

Activation of ACLY by SEC63 deploys metabolic reprogramming to facilitate hepatocellular carcinoma metastasis upon endoplasmic reticulum stress

Chenyu Hu[1], Zechang Xin[1], Xiaoyan Sun[1], Yang Hu[1], Chunfeng Zhang[2], Rui Yan[3], Yuying Wang[4], Min Lu[5], Jing Huang[6], Xiaojuan Du[4], Baocai Xing[1, *] and Xiaofeng Liu[1, *]

[1]Hepatopancreatobiliary Surgery Department I, Key laboratory of Carcinogenesis and Translational Research (Ministry of Education/Beijing), Peking University Cancer Hospital & Institute, Beijing 100142, P. R. China

[2]Department of Medical Genetics, School of Basic Medical Sciences, Peking University Health Science Center, Beijing 100191, P. R. China

[3]Department of Genetics, Harvard Medical School, Boston 02115, USA

[4]Department of Cell Biology, School of Basic Medical Sciences, Peking University Health Science Center, Beijing 100191, P. R. China

[5]Department of Pathology, School of Basic Medical Sciences, Peking University Health Science Center, Beijing 100191, P. R. China

[6]Department of Immunology, School of Basic Medical Sciences, Peking University, and NHC Key Laboratory of Medical Immunology (Peking University), Beijing 100191, P. R. China

Correspondence:

Xiaofeng Liu and Baocai Xing, Hepatopancreatobiliary Surgery Department I, Key laboratory of Carcinogenesis and Translational Research (Ministry of Education/Beijing), Peking University Cancer Hospital & Institute, Beijing 100142, P. R. China

Email: liuxiaofeng100@bjmu.edu.cn; xingbaocai88@sina.com.

Supplementary Figures

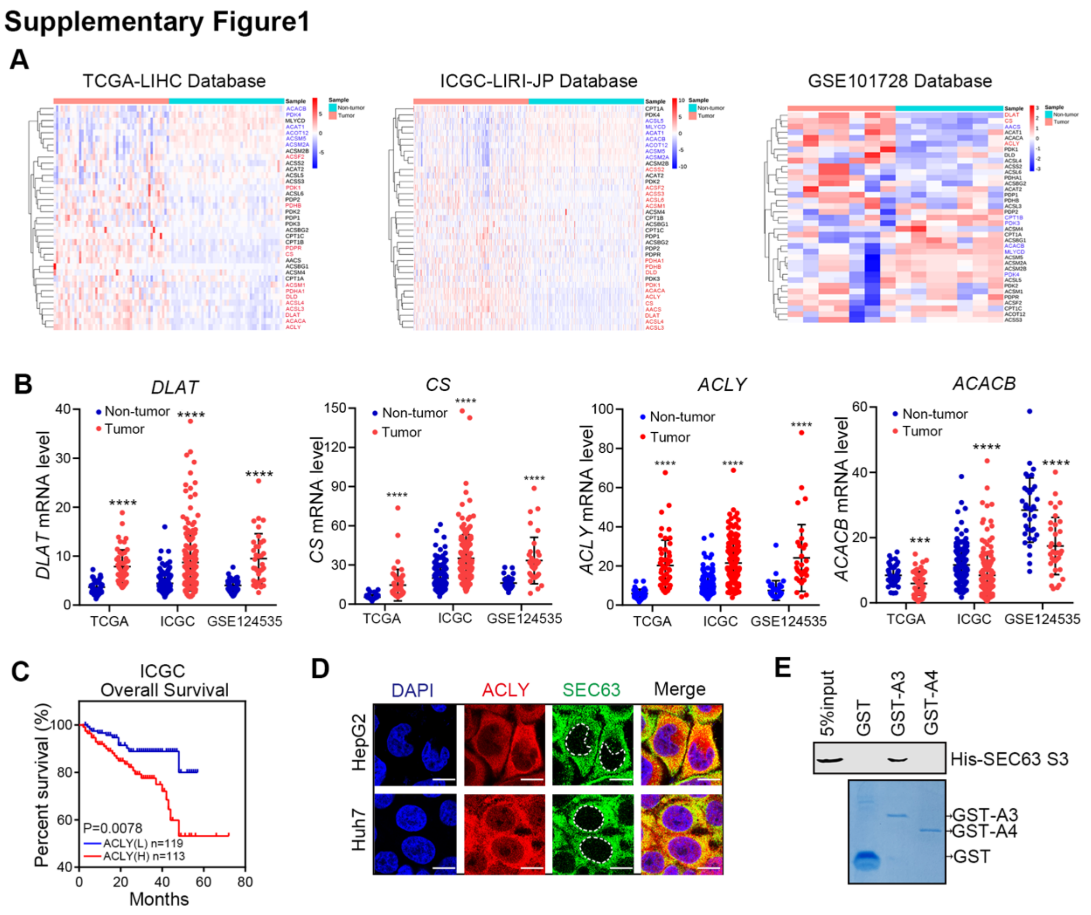

Figure S1. ACLY is upregulated by SEC63 in response to ER stress in HCC cells. **A** The heatmaps displaying the differentially expressed ARGs from TCGA-LIHC, ICGC-LIRI-JP, and GSE101728 cohorts. **B** The expression level of the indicated genes was analyzed in the indicated cohorts. **C** The overall survival curve of the HCC patients with different ACLY mRNA levels from ICGC was analyzed by Kaplan-Meier analyses. **D** Triple immunofluorescene (IF) staining for ACLY (red), SEC63 (green), and nuclei (DAPI, blue) was performed in Huh7 and HepG2 cells. Scale bar, 10 μm. **E** GST pulldown assay with purified fragments as indicated.

Supplementary Figure2

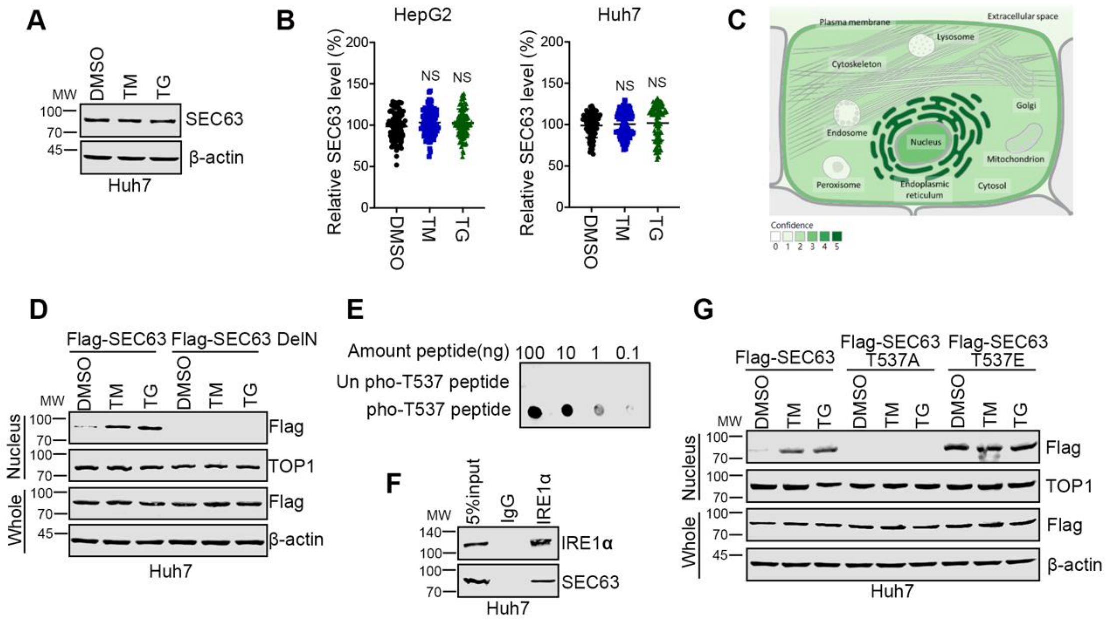

Figure S2. SEC63 is phosphorylated at T537 after ER stress. **A** Western blot analysis of SEC63 in cell lysates of Huh7 cells treated as indicated. **B** Relative SEC63 expression levels in HepG2 and Huh7 cells were determined by immunofluorescence assay using Leica LAS X confocal software (n = 100). **C** The predication analysis of SEC63 localization by COMPARTMENTS. **D** Cell fraction was performed and western blot was employed for analyzing the indicated protein. **E** Dot blot analysis was performed for validating T537-p antibody. **F** Cell lysates from Huh7 cells were subjected to Co-IP using the indicated antibody. **G** Cell fraction was performed and the indicated proteins were determined by western blot.

Supplementary Figure3

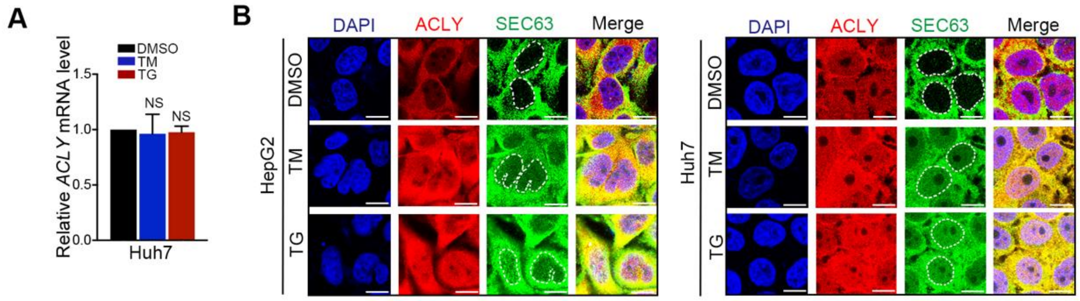

Figure S3. SEC63 increases the stability of ACLY. **A** Total RNA extracted from the Huh7 cells treated with TM (5 μg/mL) or TG (1 μM) were subjected to RT-qPCR as indicated. **B** HepG2 and Huh7 cells were treated with TM (5 μg/mL) or TG (1 μM) and immunofluorescence was performed. Scale bar, 10 μm.

Supplementary Figure4

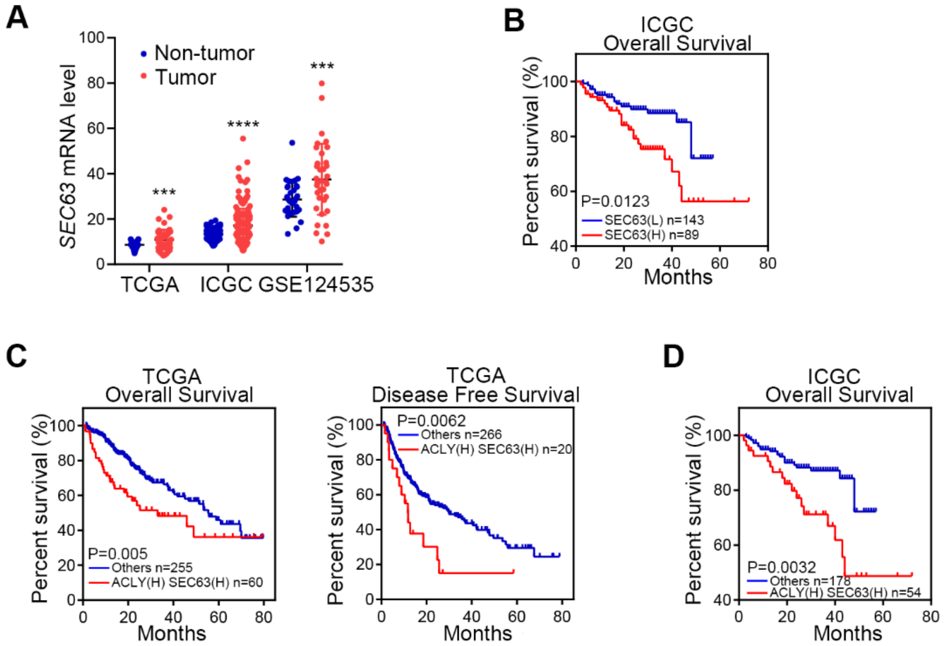

Figure S4. SEC63 is associated with prognosis of HCC patients. **A** The mRNA level of SEC63 was analyzed as indicated. **B** The overall survival curve of the HCC patients with different SEC63 mRNA levels from ICGC database was analyzed by Kaplan-Meier analyses. **C** The overall survival and disease-free survival curves of HCC patients were plotted against the SEC63 and/or ACLY mRNA levels based on the prognosis data of TCGA-LIHC database using the Kaplan-Meier method. The difference between the survival curves was analyzed using the log-rank test. **D** Overall survival curve of HCC patients was plotted against the SEC63 and/or ACLY mRNA levels based on the prognosis data of ICGC database using the Kaplan-Meier method. The difference between the survival curves was analyzed using the log-rank test.

Supplementary Figure5

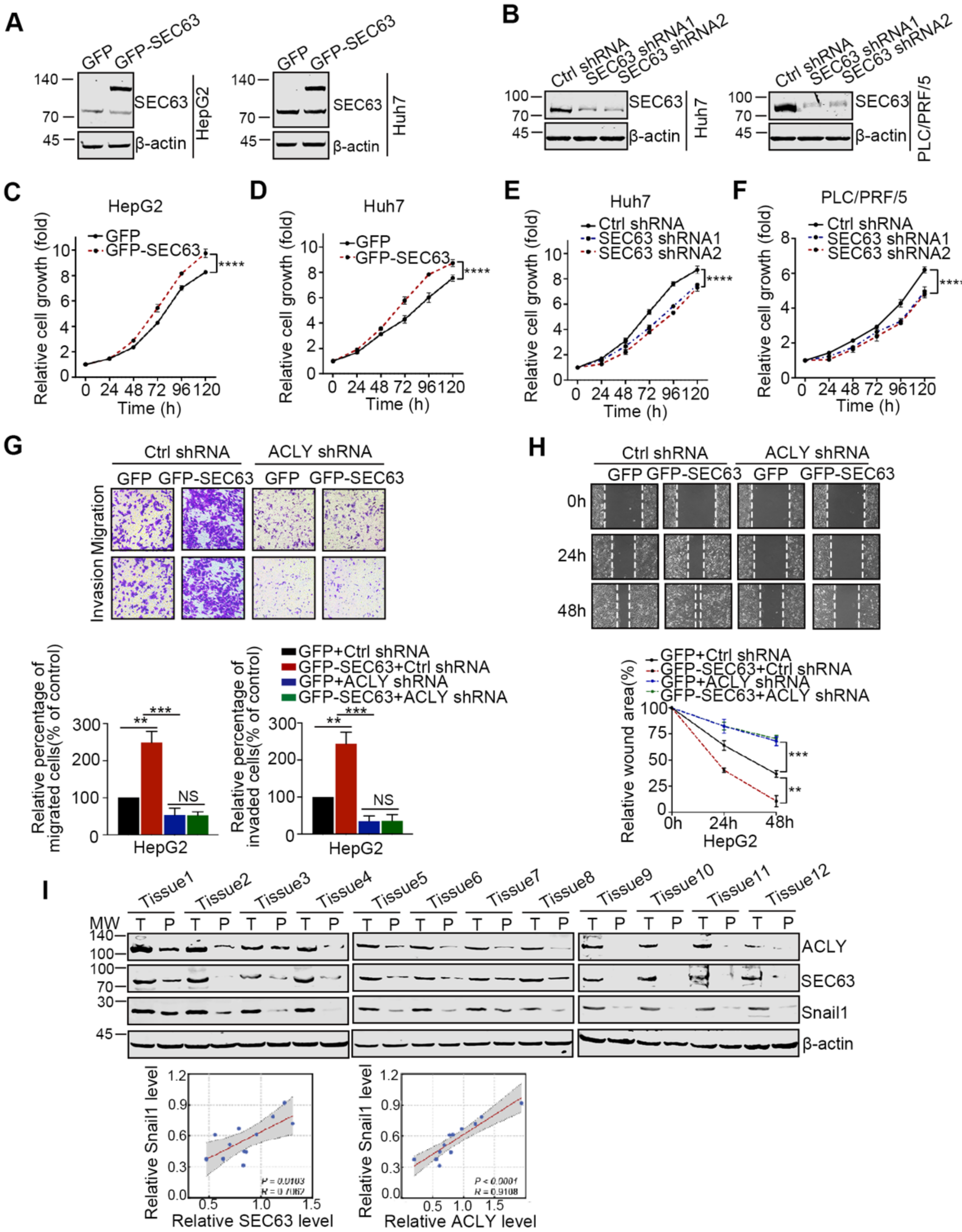

Figure S5. SEC63 promotes HCC cell metastasis relying on ACLY. **A** HepG2 or Huh7 stable cell lines with overexpression of SEC63 were generated. Western blot was performed for analyzing the expression level of SEC63. **B** Huh7 or PLC/PRF/5 stable cell lines with knockdown of SEC63 were generated. The knockdown effect was determined by western blot. **C-F** The cells were treated as indicated and cell proliferation was assessed by MTT assay. **G** Migration assay or invasion assay was performed as indicated. Migration or invasion rate was further quantified (n = 3). **H** Wound healing assay was performed as indicated. Wound closure rate was quantified (n = 3). **I** Western blot analysis of the indicated proteins in 12 individual paired HCC tissues used in Figure 4I.

Supplementary Figure6

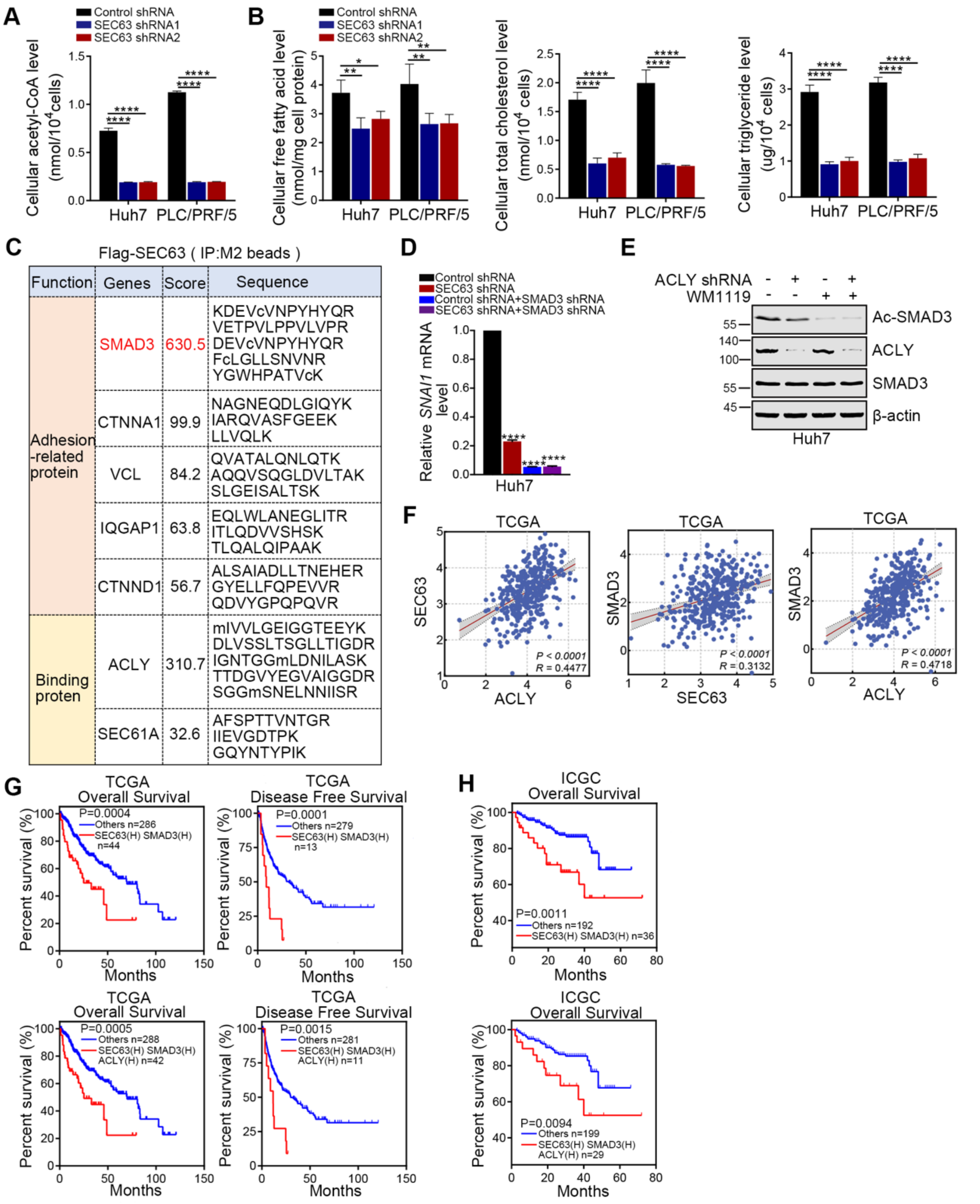

Figure S6. SEC63 modulates ACLY-related metabolic and epigenetic reprogramming. **A** The cellular acetyl-CoA level was evaluated in the indicated cells (n = 4). **B** The cellular free fatty acids, cholesterol level, and triglyceride levels were evaluated in the indicated cells (n = 4). **C** The potential interacting proteins of SEC63 related to ER stress were identified by liquid chromatography-tandem mass spectrometry. **D** Huh7 cells were transfected as indicated. Then, total RNA was extracted and RT-qPCR was performed using the *SNAI1* primers (n = 3). **E** The ctrl cells or ACLY-depleted cells were treated with WM1119. Western blot was performed for the indicated proteins. **F** The correlation analyses were analyzed as indicated using TCGA HCC dataset. **G** The overall survival or disease-free survival curves were analyzed as indicated using TCGA HCC dataset. **H** The overall survival curve was plotted as indicated using ICGC dataset.

Supplementary tables

Table S1. Correlation of SEC63/ACLY expression with clinicopathologic status in 139 cases of patients with HCC

Features	Total, n	Relative SEC63 expression		p value	Relative ACLY expression		p value
		Low(n=63)	High(n=76)		Low(n=72)	High(n=67)	
**Gender**							
Male	111	51	60	0.769	55	56	0.291
Female	28	12	16		17	11	
**Age**							
≤ 50	42	19	23	0.989	23	19	0.645
> 50	97	44	53		49	48	
**AFP**							
≤ 20	58	34	24	0.008	37	21	0.017
> 20	81	29	52		35	46	
**HBV**							
Positive	120	53	67	0.491	60	60	0.286
Negative	19	10	9		12	7	
**Liver cirrhosis**							
Yes	117	51	66	0.344	58	59	0.226
No	22	12	10		14	8	
**Tumor size(cm)**							
≤ 5	68	31	37	0.951	40	28	0.105
> 5	71	32	39		32	39	
**Intrahepatic metastasis**				0.001			0.01
Yes	36	8	28		12	24	
No	103	55	48		60	43	
**Vascular invasion**							
Yes	48	10	38	0.000	10	38	0.000
No	91	53	38		62	29	
**Lymph node metastasis**				0.000			0.000
Yes	33	2	31		2	31	
No	106	61	45		70	36	
**Edmondson grade**							
I/II	82	41	31	0.004	43	29	0.053
III/IV	67	22	45		29	38	
**BCLC stage**							
0/A	68	48	20	0.000	53	15	0.000
B/C	71	15	56		19	52	

[a] According to the immunoreactive scores from immunohistochemistry of tissue microarray; Low, (0-5), high (6-12).

[b] P values calculated using the chi-square test.

Table S2. Reagents and antibodies

Reagent	Company	Cat. No.
DMSO	Sigma -Aldrich	D2650
DMEM	Thermo Fisher Gibco	11995040
RPMI 1640	Thermo Fisher Gibco	11875119
Fetal bovine serum	Thermo Fisher Gibco	16140071
Glutathione sepharose 4B	GE Healthcare, Piscataway, NJ	17-0756-01
Tunicamycin	MedChemExpress	HY-A0098
Thapsigargin	MedChemExpress	HY-13433
MG132	Sigma -Aldrich	M8699
Cycloheximide	Sigma -Aldrich	R750107
KIRA6	Selleck Chemicals	S8658
WM1119	Selleck Chemicals	S8776
Bempedoic acid	MedChemExpress	HY-12357
**Antibody**	**Company**	**Cat. No.**
anti-SEC63	Abcam	Ab244497
anti-SEC63	Proteintech	67352-1-Ig
anti-ACLY	abclonal	A3719
anti-Snail1	abclonal	A11794
anti-ubiquitin	Cell Signalling Technology	3936S
anti-acetyl-H3	Active Motif	39040
anti-E-cadherin	Abcam	ab40772
anti-Vimentin	abclonal	A19607
anti-GPR78	abclonal	A11366
anti-IRE1α	abclonal	A17940
anti-DLAT	abclonal	A8814
anti-CS	abclonal	A5713
anti-TOPI	abclonal	A12409
anti-α-tubulin	abclonal	AC012
anti-KLHL25	Proteintech	27482-1-AP
anti-pan phosphor-Serine/Threonine	abclonal	AP0893
anti-phos-T537-Sec63	This study	
anti-Flag	Sigma-Aldrich	F3165
anti-Flag	TransGen Biotech	HT201
anti-GFP	TransGen Biotech	HT801
anti-β-actin	abclonal	AC026
IRDye® 680RD Goat anti-Rabbit IgG (H+L)	LI-COR Biosciences	926-68071
IRDye® 800RD Goat anti-Mouse IgG (H+L)	LI-COR Biosciences	926-32210

Table S3. shRNA sequence

Gene	Sequence
SEC63 shRNA#1	CCCTTGAAGAAGATCAGCAAT
SEC63 shRNA#2	GCCCTACTTCAAGAAATGGTT
ACLY shRNA	CTCAAGATACTATACATTT
Snail1 shRNA	AGGCTCGAAAGGCCTTCAA
SMAD3 shRNA	GCGTGAATCCCTACCACTA

Table S4. Primer used for RT-qPCR

Targets	**Primer Sequence**
ACLY	F: ACGCCCCTGAAGACAAGAAA
	R: CAAGGGGATTGATCTCGAGG
SEC63	F: CGAGCAAATTCGATTAAAGAATATC
	R: CCTGCAAGCAGAACTATTTTCTTTA
Snail1	F: CTAGGCCCTGGCTGCTACAAG
	R: AGCGGGGACATCCTGAGCA
E-cad	F: CCAGAAACGGAGGCCTGAT
	R: CTGGGACTCCACCTACAGAAAGTT
Vimentin	F: GAGAACTTTGCCGTTGAAGC
	R: GCTTCCTGTAGGTGGCAATC
HSPA5	F: ACGGCAGCTGCTATTGCTTA
	R: TCCATGACACGCTGGTCAAA
HSP90B1	F: CGGTCAGAGCTGACGATGAA
	R: TAACTTCGGCTTGGAAGGCA
FASN	F: GAAGCTCGTGTTGACTTCTC
	R: AGAAGACCACAAAGTAGTCC
SCD1	F: GCAGCCGAGCTTTGTAAGAG
	R: GTTCTACACCTGGCTTTGGG
GAPDH	F: ACCACAGTCCATGCCATCAC
	R: TCCACCACCCTGTTGCTGTA
β-actin	F: ATGGGTCAGAAGGATTCCTATGT
	R: AAGGTCTCAAACATGATCTGGG
HSPA5-ChIP	F: GAACCTTGAACGGCAAGAACTTGAT
	R: GTCCTATGTCGCCTTCACTCCTGAA
HSP90B1-ChIP	F: TGACTGCCTCCGTATTTCAGGATCT
	R: AGCTCTGACCGACCCTGAAGATTTA
FASN -ChIP	F: GATAGCCTATGCTCTGGGGG
	R: CTCCTGTGGTGTGTGGGTTG
SCD1-ChIP	F: GGAGGAGATAAGTTGGAGACGATGC
	R: AAAGCCAGGTGTAGAACTTGCAGGT
SNAI1-ChIP	F: GGCCAGCAGCCGGCGCACCT
	R: GCGCAGAAGAACCACTCGCT
